# Genomes of early-diverging streptophyte algae shed light on plant terrestrialization

**DOI:** 10.1038/s41477-019-0560-3

**Published:** 2019-12-16

**Authors:** Sibo Wang, Linzhou Li, Haoyuan Li, Sunil Kumar Sahu, Hongli Wang, Yan Xu, Wenfei Xian, Bo Song, Hongping Liang, Shifeng Cheng, Yue Chang, Yue Song, Zehra Çebi, Sebastian Wittek, Tanja Reder, Morten Peterson, Huanming Yang, Jian Wang, Barbara Melkonian, Yves Van de Peer, Xun Xu, Gane Ka-Shu Wong, Michael Melkonian, Huan Liu, Xin Liu

**Affiliations:** 1 BGI-Shenzhen, Shenzhen, China; 2 China National GeneBank, BGI-Shenzhen, Shenzhen, China; 3 Department of Biology, University of Copenhagen, Copenhagen, Denmark; 4 State Key Laboratory of Agricultural Genomics, BGI-Shenzhen, Shenzhen, China; 5 Department of Biotechnology and Biomedicine, Technical University of Denmark, Lyngby, Denmark; 6 BGI Education Center, University of Chinese Academy of Sciences, Shenzhen, China; 7 Botanical Institute, Cologne Biocenter, University of Cologne, Cologne, Germany; 8 Department of Plant Biotechnology and Bioinformatics, Ghent University and VIB/UGent Center for Plant Systems Biology, Ghent, Belgium; 9 Centre for Microbial Ecology and Genomics, Department of Biochemistry, Genetics and Microbiology, University of Pretoria, Pretoria, South Africa; 10 Department of Biological Sciences and Department of Medicine, University of Alberta, Edmonton, Alberta Canada; 11 Present Address: University of Duisburg-Essen, Campus Essen, Faculty of Biology, Essen, Germany

**Keywords:** Phylogenetics, Plant evolution

## Abstract

Mounting evidence suggests that terrestrialization of plants started in streptophyte green algae, favoured by their dual existence in freshwater and subaerial/terrestrial environments. Here, we present the genomes of *Mesostigma viride* and *Chlorokybus atmophyticus*, two sister taxa in the earliest-diverging clade of streptophyte algae dwelling in freshwater and subaerial/terrestrial environments, respectively. We provide evidence that the common ancestor of *M. viride* and *C. atmophyticus* (and thus of streptophytes) had already developed traits associated with a subaerial/terrestrial environment, such as embryophyte-type photorespiration, canonical plant phytochrome, several phytohormones and transcription factors involved in responses to environmental stresses, and evolution of cellulose synthase and cellulose synthase-like genes characteristic of embryophytes. Both genomes differed markedly in genome size and structure, and in gene family composition, revealing their dynamic nature, presumably in response to adaptations to their contrasting environments. The ancestor of *M. viride* possibly lost several genomic traits associated with a subaerial/terrestrial environment following transition to a freshwater habitat.

## Abstract (web summary)

A study sequenced and analysed two genomes of basal streptophyte algae that adapt to freshwater and subaerial/terrestrial environments, respectively, providing insights into plant terrestrialization, an important evolutionary transition in the history of life.

## Main

Transition to a terrestrial environment, termed terrestrialization, is generally regarded as a pivotal event in the evolution and diversification of land plant flora^1^. Extant green plants (Viridiplantae) can be subdivided into two lineages, Chlorophyta (most of the green algae) and Streptophyta (embryophytes and their closest algal relatives, a grade collectively known as streptophyte algae^2^). There is now compelling evidence that adaptation to subaerial/terrestrial habitats is a feature of streptophyte algae, arising from their dual existence in freshwater and subaerial/terrestrial environments throughout their evolutionary history. Recent transcriptomic and genomic studies have shown that the molecular toolkit for life in a terrestrial environment was already present in streptophyte algae^3^. Homologues of genes once thought to be restricted to embryophytes are now being detected in streptophyte algae. Examples include those involved in symbiotic or pathogenic interactions with soil microbes^4^, phytohormone signalling^5–8^, desiccation/stress^9^, plastid/nucleus retrograde signalling^10,11^ and cell wall metabolism^12^. Importantly, many transcription factors (TFs) thought to be specific to embryophytes originated in streptophyte algae and also substantially expanded there^13^. These findings raise exciting questions about the functional role of embryophyte-like genes in streptophyte algae.

**Fig. 1:**
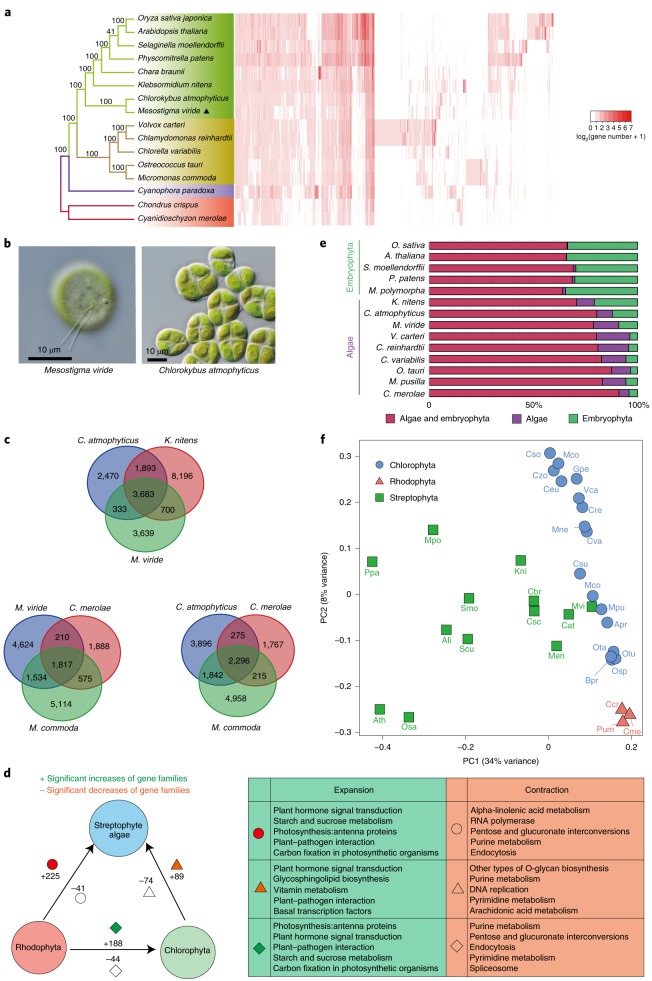
Comparative genome profile of *M. viride* and *C. atmophyticus*. **a**, This phylogenetic tree was constructed by maximum likelihood based on the concatenated sequences of single-copy genes, while species-specific gene duplicates were excluded from the analysis. A *k*-means clustering of gene families based on the gene abundance of each species is shown in the right-hand panel; each column represents a family and each row represents one species. **b**, Differential interference contrast micrographs showing *M. viride* (left) and *C. atmophyticus* (right). **c**, Venn diagrams showing the number of gene families shared among *M. viride*, *C. atmophyticus* and a representative rhodophyte, streptophyte or chlorophyte. **d**, Significant increases and decreases in gene families; filled red circles, triangles and rhombi denote function enrichment of significant increased gene families in the KEGG pathway, while empty symbols denote function enrichment of significant decreased gene families in the KEGG pathway. Details of these functions are shown in the right-hand panel. **e**, Percentages of total proteins found in both algae and embryophytes (red), proteins shared among algae (purple) and proteins shared among embryophytes (green) based on the classification given in Orthofinder. **f**, Principal component analysis of the type and number of Pfam domains.

In phylogenomic analyses, the earliest-diverging streptophyte algae are represented by a clade comprising two monospecific genera, *Mesostigma* and *Chlorokybus*^14–17^. Both are structurally simple but differ in their cellular organization, life history and type of habitat^18^. *Mesostigma viride* is a scale-covered flagellate with an eyespot that reproduces by binary division at the flagellate stage (Fig. 1b). *Chlorokybus atmophyticus* consists of sarcinoid cell packets where each cell has its own cell wall, occasionally producing biflagellate, scaly zoospores (Fig. 1b). Importantly, *M. viride* is found in the benthos of small, shallow ponds whereas *C. atmophyticus* is a subaerial/terrestrial alga that occurs among bryophytes, on soil and on stones.

Previous analyses of two streptophyte algal genomes, *Klebsormidium nitens*^6^ and *Chara braunii*^7^, have not only revealed embryophyte-like genomic traits with gains and expansions of respective genes/gene families in both taxa, but also loss of genes involved in responses to abiotic stresses that prevail in a terrestrial environment, in the aquatic (freshwater) *C. braunii* (*K. nitens* thrives in a subaerial/terrestrial environment). The draft genomes of *M. viride* and *C. atmophyticus* reported here allowed us to address two questions important for plant terrestrialization: (1) did the common ancestor of streptophytes already display embryophyte-like genomic traits that would be indicative for adaptation to a terrestrial environment; and (2) how do the genomes of *M. viride* and *C. atmophyticus* differ from each other in light of previous genome studies on two streptophyte algae that occur in contrasting environments (subaerial/terrestrial and aquatic) but belong to different streptophyte classes (Klebsormidiophyceae and Charophyceae)?

## Results

### Genome sequencing, genome characteristics and phylogenetic analysis

A total of 245 Gb (746.86X) (*M. viride*) and 66 Gb (775.68X) (*C. atmophyticus*) raw data were generated using Illumina technology (Supplementary Tables 1 and 2). Based on *k*-mer depth distribution analyses, the nuclear genome sizes of *M. viride* and *C. atmophyticus* were estimated to be 329 and 85 Mb, respectively (Supplementary Tables 3 and 4 and Supplementary Figs. 1 and 2). Approximately 85.4% (281 Mb) and 87.0% (74 Mb) of the genomes were de novo assembled, consisting of 6,924 scaffolds with a minimum contig length needed to cover 50% of the genome (*N*_50_) of 113,221 base pairs (bp) for *M. viride*, and 3,836 scaffolds with *N*_50_ of 752,385 bp for *C. atmophyticus* (Supplementary Tables 5 and 6). The distributions of genomic copy content (Supplementary Figs. 3 and 4) and BUSCO score (Supplementary Tables 7 and 8) suggested no contamination and good assembly quality. Moreover, 93.8% (*M. viride*) and 99.4% (*C. atmophyticus*) of de novo assembled transcripts were aligned to the assembled genome. (Supplementary Tables 9 and 10). Both genomes have substantial repeat components (38.7 and 31.3%, respectively; Supplementary Tables 11 and 12). By combining homologue-based, ab initio and transcriptome-based approaches, 9,300 gene models were predicted for both genomes (Supplementary Tables 13 and 14 and Supplementary Fig. 5). In total, 9,198 and 9,066 predicted coding sequences, respectively, were supported by sequenced transcripts, indicating the high accuracy of gene predictions (Supplementary Table 15). Complete coverage of genes involved in transcription, translation and DNA synthesis was also obtained (Supplementary Table 16).

A phylogenetic analysis of 16 genomes from Rhodophyta, Glaucophyta, Chlorophyta, streptophyte algae and embryophytes, based on a concatenated amino acid sequence alignment of 375 orthologues of single-copy genes, confirmed the sister relationship between *Mesostigma* and *Chlorokybus* and also supported previous reports that *M. viride* and C*. atmophyticus* constitute the earliest-diverging lineage of extant streptophytes^15–17^ (Fig. 1a).

### Comparative genomics

**Extended Data Fig. 1:**
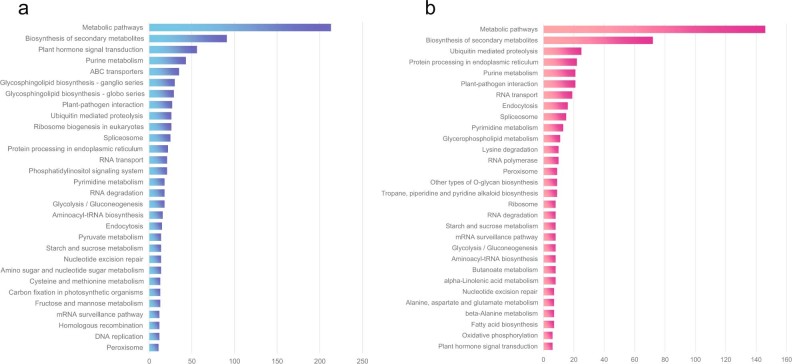
The KEGG distribution of unique proteins in M. viride (blue) and C. atmophyticus (pink). The x-axis indicates the number of genes in a specific category in the respective species. The metabolism pathway is shown on the y-axis.

*Mesostigma viride*, *C. atmophyticus* and *K. nitens* shared 3,683 gene families (Fig. 1c), which comprised 4,423 genes in *M. viride and* 4,210 in *C. atmophyticus*. Interestingly, 3,639 and 2,470 gene families were found exclusive to *M. viride* and *C. atmophyticus*, respectively (Extended Data Fig. 1 and Supplementary Table 17). We also considered homologues of the identified genes in the red alga *Cyanidioschyzon merolae*, and in *Micromonas commoda* (Chlorophyta). *M. viride* and *C. atmophyticus* share about 2,000–2,500 gene families with *C. merolae and* 3,300–4,100 with *M. commoda*, reflecting their phylogenetic relationships (Fig. 1c).

To explore significant increases or decreases in gene families in Rhodophyta, Chlorophyta and streptophyte algae, the gene family content of five representative genomes in each of the three clades (a total of 15 genomes) was compared. The most significant increases in gene families occurred from Rhodophyta to streptophyte algae (225) and to Chlorophyta (188), respectively, and referred mainly to plant hormone signal transduction, plant–pathogen interaction, photosynthesis and starch and sucrose metabolism (Fig. 1d). Conversely, significant decreases in gene families were observed from the two lineages of Viridiplantae to Rhodophyta (41 and 44, respectively), mainly corresponding to purine metabolism, endocytosis and pentose and glucuronate interconversions. In comparison, significant increases and decreases in gene families between streptophyte algae and Chlorophyta were more modest: the main increases for gene families in streptophyte algae were in plant hormone signal transduction, plant–pathogen interactions and TFs, whereas the main decreases for gene families were in purine and pyrimidine metabolism, DNA replication and O-glycan biosynthesis.

In addition, our analysis revealed a higher percentage of embryophyte genes in *M. viride* and *C. atmophyticus* compared to Chlorophyta, but lower than in *K. nitens* (Fig. 1e). In addition to gene families, the total number of conserved Pfam domains in Rhodophyta, Chlorophyta and Streptophyta was subjected to principal component analysis, which showed distinct patterns of functional diversification indicating evolutionary diversification (Fig. 1f). Overall, these results suggest that the genomes of early-diverging streptophyte algae already contained archetypal genes that typically exist in modern embryophytes.

### TFs and phytohormones in early-diverging Streptophyta

**Fig. 2:**
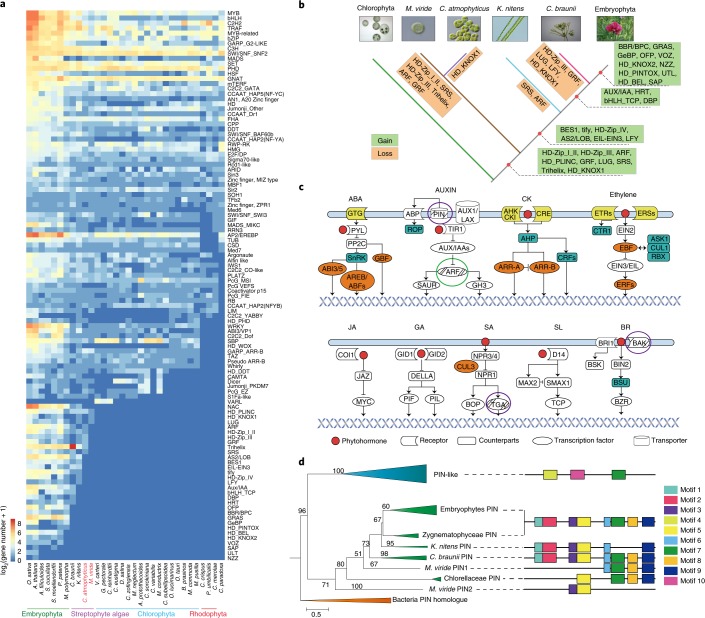
Analysis of TF genes and phytohormone signalling pathways in *M. viride* and *C. atmophyticus*. **a**, Using a HMMER approach for the respective genomes, the numbers of TFs and TRs were identified using the TAPscan database v.2 (for details see Methods). **b**, Illustrative phylogenetic representation of the predicted gain (green) and loss (orange) of plant TFs in streptophyte algae. **c**, Presence/absence of the main phytohormone signalling pathways deduced from the genomes of *M. viride* and *C. atmophyticus*. Coloured boxes indicate the presence of genes in the pathways, white boxes their absence. All searches were done using HMM (1 × 10^–10^). Purple-lined ellipses denote genes identified in *M. viride* but not in *C. atmophyticus*, while the green-lined ellipse denotes a gene identified in *C. atmophyticus* but not in *M. viride*. **d**, The maximum-likelihood method was used to draw the phylogenetic tree of the PIN and PIN-related homologues to understand their origin among Streptophyta.

Out of 114 types of TFs/transcription regulators (TRs) analysed, 72 (*M. viride)* and 80 (*C. atmophyticus)* TFs/TRs were identified in the two genomes (Fig. 2a and Supplementary Table 18), of which most are associated with abiotic stress responses, development and plant–pathogen interactions in embryophytes. TF/TR genes accounted for 3.31% of the total number of protein-coding genes in the *M. viride* genome and 4.47% in the *C. atmophyticus* genome, similar to the percentages in bryophytes (4.68%) and angiosperms (~5%). A combination of HMMER and phylogenetic analyses (for details see Methods) revealed putative gains of several TF/TR genes in the common ancestor of streptophytes, and differential losses of some of these genes in *M. viride* and/or *C. atmophyticus* (Fig. 2b). We cannot, however, exclude the possibility that the lower TF/TR numbers in *M. viride* relate to lack of representation in the genome assembly, although we aimed to compensate for this by adding data from our deeply sequenced transcriptome. Among the gains, the homeodomain–leucine zipper (HD-ZIP) family members are unique to plants and have diverse functions in growth and development, mostly related to stress responses^19^. Of the four known classes (I–IV) of HD-ZIPs, gene classes I–III were found in *C. atmophyticus* but were missing in *M. viride* (Fig. 2a and Supplementary Fig. 6). *M*. *viride* displayed one gene that was positioned near the base of the HD-ZIPIV clade in phylogenetic analyses with moderate support and a long branch (Supplementary Fig. 6). However, this gene contained neither a HD nor a START domain, suggesting that it represents either an ancestral pre-HD-ZIP_IV or a degenerated gene with domain, and perhaps functional, loss. Other TF/TR genes that apparently originated in the common ancestor of streptophytes, such as auxin response factors (ARF) proto-C-type (Fig. 2b and Supplementary Figs. 7 and 8), SHI-related sequence (SRS), Trihelix TF family (Trihelix), growth-regulating factors (GRF), LUG transcriptional co-repressor (LEUNIG) and HD-PLINC homeodomain plant zinc finger (HD-PLINC), have also been implicated in the regulation of growth and development in response to light, salt and pathogen stresses in embryophytes^20–25^. In addition to gains, nine TF/TR gene families expanded by a minimum of threefold in *M. viride*/*C. atmophyticus* compared to non-streptophytes (Supplementary Table 18), and five of those (C2C2_CO-like, DDT, PcG_FIE, PcG_MSI, PSEUDO-ARR-B) had a minimum of 50% more members in *C. atmophyticus* than in *M. viride*, all involved in responses to abiotic and biotic stresses in embryophytes. Overall, our results correlate well with a recent analysis using transcriptome data^13^, except that we extend the origin of HD-Zip genes I–III, ARF and HD-KNOX1 (Knotted-like homebox class I) to the common ancestor of streptophytes rather than a later origin.

Next, we explored phytohormone biosynthesis and transduction pathway-related genes (Fig. 2c and Supplementary Tables 19 and 20). By employing a HMMER search, an almost intact abscisic acid (ABA) signalling pathway was detected although the ABA receptor PYL was absent, the latter finding consistent with earlier reports on *K. nitens*^6^ and *C. braunii*^7^. Homologues of the Abscisic acid responsive element-binding protein (AREB)/Abscisic acid responsive element-binding factors (ABFs) TFs involved in ABA signalling under drought stress in embyophytes were also detected in both genomes^26^. Genes for the complete signalling pathway of cytokinin (CK^27^) were identified in both genomes. The second, conserved, two-component signalling pathway (in addition to CK), that of ethylene, was also almost completely represented except for the TF EIN3/EIL.

Both *M. viride* and *C. atmophyticus* were found to encode various transporter proteins and ATP-binding cassette B transporters that could potentially participate in auxin transport (Supplementary Table 21). However, no auxin receptor (TIR) was found in either genome (Fig. 2c). Notably, PIN, an important auxin efflux carrier involved in the transport of auxin between cells, was detected in the *M. viride* genome but not in *C. atmophyticus*. We found a step-by-step gain in motifs corresponding to PIN (Fig. 2d). Neither *M. viride* nor *C. atmophyticus* encodes the auxin signal transduction components AUX/IAA1, SAUR and GH3 (Fig. 2c). Furthermore, both genomes lacked auxin biosynthesis genes such as tryptophan-aminotransferase (TAA) and nitrilase, while YUCCA could be identified in *M. viride* only (Supplementary Table 19 and Supplementary Fig. 9).

**Extended Data Fig. 2:**
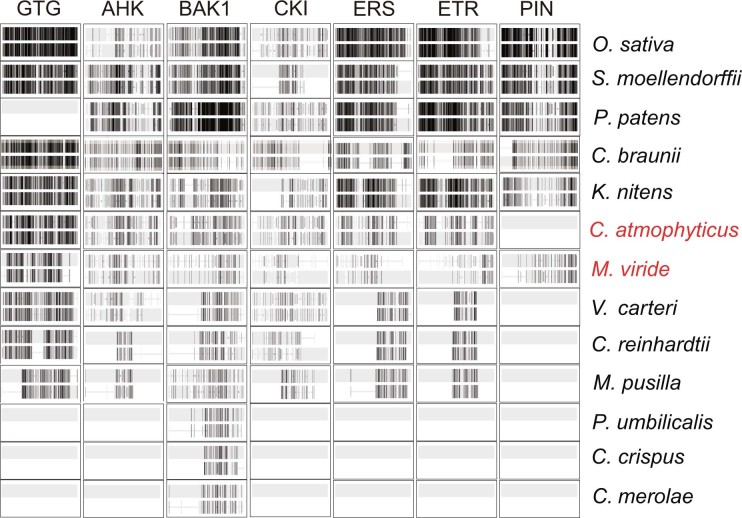
Sequence conservation of various phytohormone receptor genes in representative species. Each cell shows a pairwise sequence alignment between a known Arabidopsis protein receptor (top) and the best BLAST hit (E-value <1e−10) in the translated genome of the indicated species. Black and grey represent similar amino acids (the darker the bars, the higher is the similarity).

Both organisms could synthesize most of the jasmonic acid (JA)-precursor OPDA but lacked OPR3, indicating that the JA biosynthetic pathway may exist in early-diverging streptophyte algae (Supplementary Table 19). Concomitantly, both *C. atmophyticus* and *M. viride* lacked the complete JA and strigolactone (SL)) signalling components and the respective hormone receptors (Fig. 2c). Gibberellic acid (GA) receptors (GID1 and GID2) and signalling components (DELLA, PIL1 and PIF) were also absent (for the PIF and bHLH phylogeny, see Supplementary Figs. 10 and 11). Some components of the salicylic acid (SA) pathway (CUL3 and TGA) were, however, detected (for TGA see Supplementary Figs 12 and 13). Finally, we analysed sequence conservation of phytohormone receptor genes among representative algal lineages (Extended Data Fig. 2). The results showed that, although the genomes of *M. viride* and *C. atmophyticus* encode some phytohormone-related receptors, they are not as well conserved as in embryophytes. In general, our results are in accordance with previous genome analyses of later-diverging streptophyte algae and liverworts^5–8^ which suggested that F-box-mediated phytohormone signalling pathways (auxin, JA, GA, SL) evolved in the embryophyte ancestor, whereas two-component system phytohormone signalling (CK, ethylene) evolved much earlier, having been present in the common ancestor of streptophytes (this study). Furthermore, some components of both F-box-mediated (auxin, SA) and other phytohormone signalling pathways (ABA, brassinosteroid (BR)) are ancient and presumably originated in the common ancestor of streptophytes—for example, PIN, C-type ARF and TGA (this study).

### Analysis of cell wall metabolism, evidence of sexual reproduction and analysis of flagellar genes

In total, 81 and 100 putative carbohydrate-active enzymes (CAZymes) and 20 and 22 additional proteins containing putative carbohydrate-binding modules were identified in *M. viride* and *C. atmophyticus*, respectively (Supplementary Table 22). Both the numbers of glycosyl hydrolases (GH) and glycosyltransferases (GT) were higher in *C. atmophyticus* than in *M. viride*, but in the same range as those found in unicellular red algae (32/54) or early-diverging Chlorophyta^28^ (52/57/77). Compared to previous transcriptome-based analyses^12^, the differences in numbers of GTs between *M. viride* and *C. atmophyticus* were not as large, suggesting that hidden life history stages in *M. viride* (zygotes?) may contribute GTs to its overall genomic GT complement. The largest GT families found were GT2 (*N*-glycosylation and cell wall biosynthesis), GT5 (starch synthases) and GT35 (glycogen/starch phosphorylases). However, certain differences were also encountered: GT 77 (involved in rhamnogalacturonan-II synthesis) had more family members in *C. atmophyticus*^29^, while GT 41 (β-*N*-acetylglucosaminyltransferase) was more prominent in *M. viride*. Four GT families were present in only *M. viride* or *C. atmophyticus*, among them GT8 (in *C. atmophyticus*), a large family of glycosyltransferases in embryophytes, of which some members have been implicated in responses to abiotic stress^30^.

**Fig. 3:**
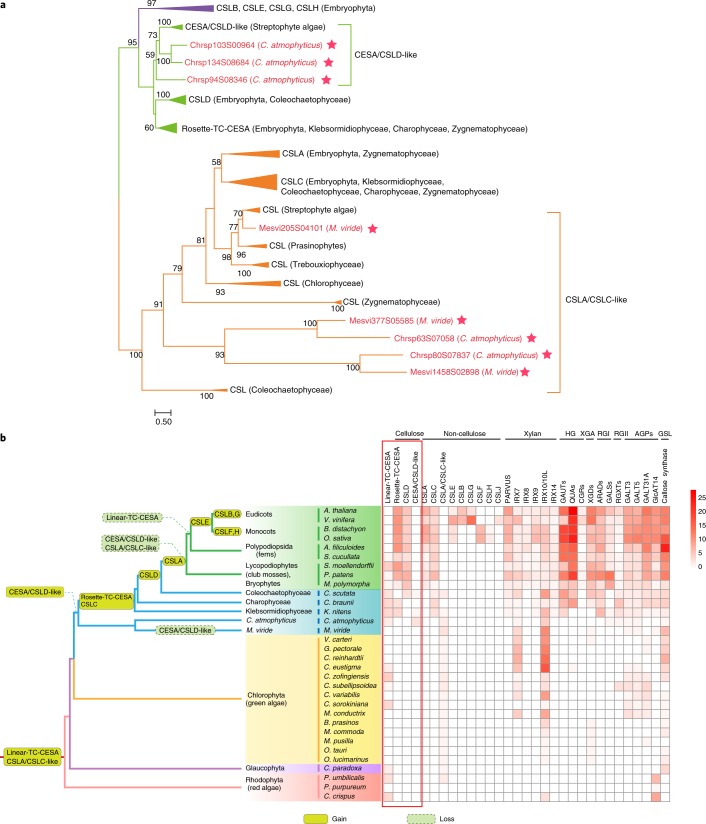
Phylogenetic distribution of enzymes involved in cell wall biosynthesis in *M. viride* and *C. atmophyticus*. **a**, Phylogenetic tree of CESA and CSL (GT2 family). The tree was constructed using the maximum-likelihood method. **b**, Left: summary of the gains and losses of cell wall biosynthetic genes mapped on the phylogenetic tree. Right: copy number of the respective cell wall biosynthetic genes. HG, homogalacturonan; XGA, xylogalacturonan; RGI and RGII, rhamnogalacturonan I and II; AGP, arabinogalactan protein; GSL, glucan synthase-like.

To gain further insight into the evolution of proteins involved in cell wall biosynthesis, we analysed the phylogeny of representative enzymes. Our analyses revealed the presence of three putative cellulose synthase-like (CSL) enzymes (CSLA/CSLC-like), but the absence of cellulose synthase (CESA), in *M. viride* (Fig. 3a and Supplementary Table 23). However, three CESA/CSLD-like homologues were identified in the *C. atmophyticus* genome and showed high similarity to the respective genes in embryophytes. *C. atmophyticus* displayed two additional CSL enzymes that apparently originated by a gene duplication in the common ancestor of *M. viride* and *C. atmophyticus* (Fig. 3a). Our phylogenetic analysis demonstrated that CESA and CSL proteins diverged into two clades, their origin apparently dating back to the ancestor of the Archaeplastida^31^. The first clade encompassed CESA and CLSB, -D, -E, -G and -H, the second clade CSLA, CSLC and a paraphyletic set of genes (CSLA/CSLC-like) that includes five CSL genes from *M. viride* and *C. atmophyticus*. Mapping CESA and CSLs on a simplified phylogenetic tree of Archaeplastida pinpoints putative gains and losses of these enzymes on different branches (Fig. 3b).

To adapt to rapidly changing environments, early-diverging streptophytes would be expected to actively conduct reassembly and degradation of cell wall components to enhance the flexibility of the cell wall during osmotic stress. Enzymes in 14 families of glycoside hydrolases (GH5-10, -12, -26, -44, -45, -48, -51, -61 and -74) are known to degrade cellulose in embryophytes^32^. Not surprisingly, cellulases were not detected in the *M. viride* genome, which lacks CESA; however, in *C. atmophyticus* cellulases were present (Table 1). Interestingly, a majority of the genes involved in mannan and xylan metabolism, such as mannanases, mannosidase and xylosidase, were detected in *M. viride* whereas *C. atmophyticus* lacks those enzymes. *M. viride* and *C. atmophyticu*s also lack xyloglucan- and xylan-degrading enzymes, as well as most of the pectin lyases (Table 1).

**Table 1:** GH families involved in degradation of cell wall components present in *M. viride*, *C. atmophyticus* and *K. nitens*

	Enzyme	*M. viride*	*C. atmophyticus*	*K. nitens*
Cellulase	Endoglucanase (GH5-10, -12, -26, -44, -45, -48, -51, -61, -74)	0	3	8
Glycosidase	GH27 (α-galactosidase)	1	2	5
	GH35 (β-galactosidase)	1	2	2
	GH36 (galactinol-sucrose galactosyltransferase)	0	2	3
	GH37 (glucan endo-1,3-β-glucosidase)	0	0	9
	GH1 (β-glucosidase)	1	1	5
Xylosidase	GH31 (α-xylosidase)	0	2	0
	GH3 (β-D-xylosidase)	1	1	2
Xyloglucan	GH12 (xyloglucan endotransglucosylase/hydrolase)	0	0	0
	GH16 (xyloglucan endotransglucosylase)	0	0	5
Xylanases	GH10 (1,4-β-xylan endohydrolase)	1	0	9
Xyloglucanase	GH77 (xyloglucanase)	0	4	3
Arabinofuranosidase	GH43 (arabinanase)	0	0	1
	GH51 (α-L-arabinofuranosidase)	1	1	1
Pectin lyases	Pectate lyase	0	0	1
	Rhamnogalacturonate lyase	0	0	1
	Pectinesterase	0	0	1
	Pectin acetylesterase	0	1	1
Mannosidase/mannanase	GH5 (mannan endo-1,4-β-mannosidase)	2	0	5

**Fig. 4:**
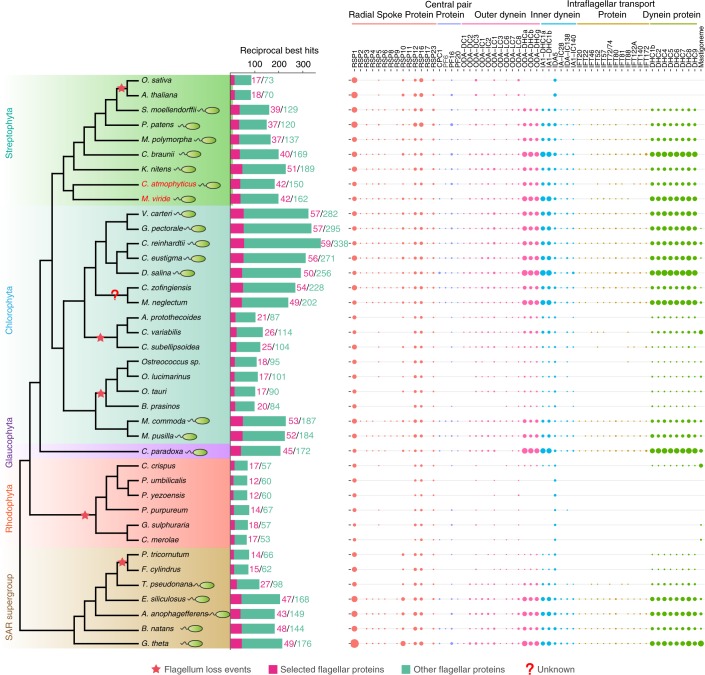
Analysis of flagellar genes and their phylogenetic distribution. The phylogenomic tree (left) was constructed using a maximum-likelihood method based on the concatenated sequences of single-copy genes from different representative algal lineages. The horizontal bar chart (middle) denotes the number of putative orthologues to 398 *Chlamydomonas* conserved flagellar proteins; the pink horizontal bar represents the number of structure-related flagellar genes (individual genes listed at the top of the right panel), while the green area represents the number of flagella-associated genes. The right panel shows the key structure-related flagellar proteins in six categories. The circle size is proportional to the copy number of putative orthologous genes found in the respective species.

**Extended Data Fig. 3:**
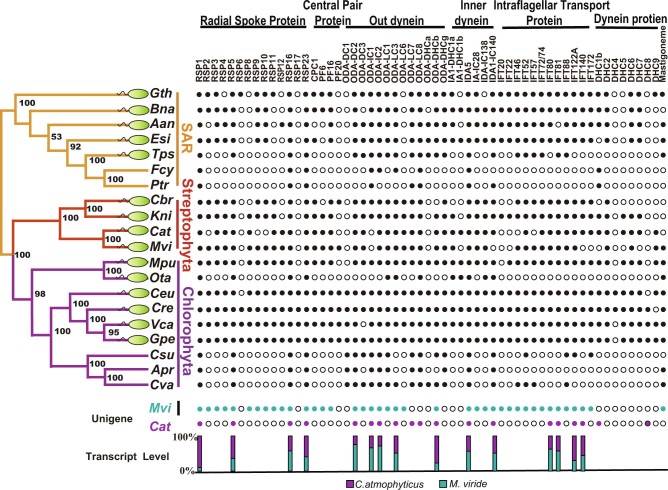
Analysis of the conserved flagellar proteome in flagellate and non-flagellate organisms and the distribution of key flagellar proteins. Key structure-related flagellar proteins in flagellate and non-flagellate algal species in different lineages. The phylogenetic tree on the left panel was constructed using maximum-likelihood method based on the concatenated sequences of single-copy genes from these genomes, after excluding the species-specific gene duplications. The presence (filled circle) or absence (empty circle) of putative orthologs to conserved flagellar proteins is shown on right panel [Based on Reciprocal Blast Hit (RBH) method with Cut-off value of e-5]. The histogram on the lower panel shows the differential expression level of these important structure-related flagellar proteins.

Some of the differences in the composition of the cell surface between *M. viride* and *C. atmophyticus* could be related to variation in their cellular organization, life history and habitats. To check whether ‘cryptic sex’ may exist in early-diverging streptophyte algae, we searched the *M. viride* and *C. atmophyticus* genomes for meiosis-specific (11) and meiosis-related genes (40) by hidden Markov model (HMM) and BLAST (Supplementary Table 24). We found that the core-set of meiosis-specific genes (10) was present in *C. atmophyticus* whereas the *M. viride* genome lacked MSH5, REC8 and RED1 (Supplementary Table 24); the absence of the latter is puzzling, but not without precedent^33^. *M. viride* reproduces vegetatively by binary division of the flagellate cell, whereas *C. atmophyticus* forms scale-covered zoospores during asexual reproduction. A comparative analysis of the complement of flagellar genes in their genomes, using a stringent reciprocal-best-BLAST-hits analysis of 397 *Chlamydomonas* flagellar proteins as query^34^, detected 204 flagellar proteins in *M. viride* and 192 in *C. atmophyticus* (Fig. 4 and Supplementary Table 25). Non-flagellate organisms lack the majority of radial spoke, as well as central pair proteins, many of the outer and inner dynein arm proteins and all intraflagellar transport proteins (IFTs), as well as dynein heavy-chain proteins. Surprisingly, in both *M. viride* and *C. atmophyticus* (flagella covered by scales), we could identify only a few IFTs whereas *K. nitens* displayed the full set (12 IFTs) (Extended Data Fig. 3).

### Evolutionary analysis of elongation factor-1α and phytochromes

**Fig. 5:**
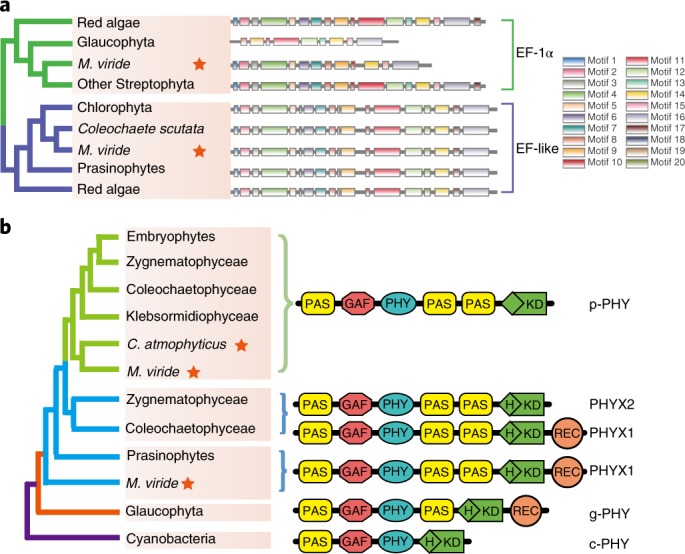
Distribution of EF-1α, EF-like and plant phytochromes. **a**, Maximum likelihood was used to infer the phylogenetic tree. Right: representative EF-1α and EF-like motifs, from red algae to embryophytes. ‘Other Streptophyta’ represents *C. atmophyticus*, Klebsormidiophyceae, Coleochaetophyceae, Charophyceae and Zygnematophyceae. **b**, Left: simplified phylogenic tree of phytochromes across cyanobacteria, glaucophytes, prasinophytes and streptophyte algae is shown. Right: complete domain structures of the phytochrome proteins. PHYX1/PHYX2 represent the sister lineage to p-PHY. The phytochromes from glaucophytes and cyanobacteria are represented by g-PHY and c-PHY, respectively.

**Extended Data Fig. 4:**
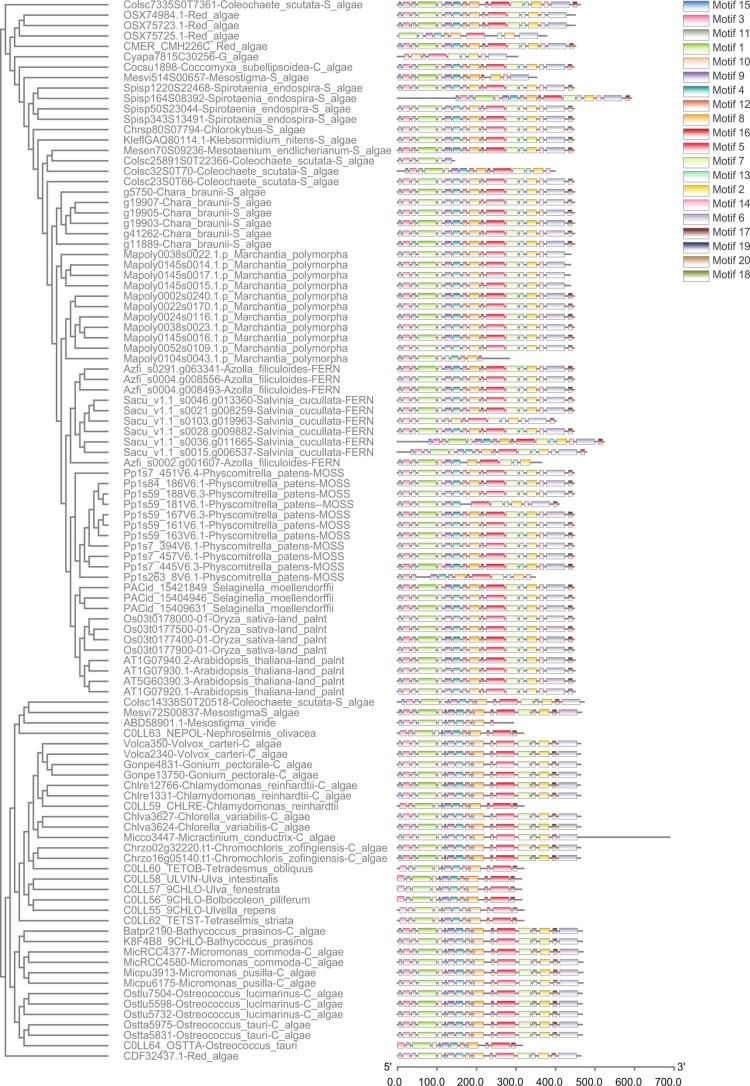
Distribution of EF-1α and EF-like motifs. Maximum Likelihood was used to infer the phylogenetic tree of the EF-1α and EF-like homologs to understand their phylogenetic distribution. The right panel displays the representative EF-1α and EF-like motifs from red algae to embryophytes. The tree derived from a MAFFT alignment and constructed using IQ-TREE (see Methods). Bootstrap values (500 replicates) ≥50% are shown.

The elongation factor EF-1α is responsible for the selection and binding of aminoacyl-transfer RNA to the A-site (acceptor site) of the ribosome. It is substituted by elongation factor-like (EF-like) proteins in many eukaryotes^35^. Intriguingly, we found that the *M. viride* genome encodes both EF-1α and EF-like genes (Fig. 5a and Extended Data Fig. 4), contradicting earlier studies that reported only the presence of EF-like genes in *M. viride* and no EF-1α (ref. ^36^).

**Extended Data Fig. 5:**
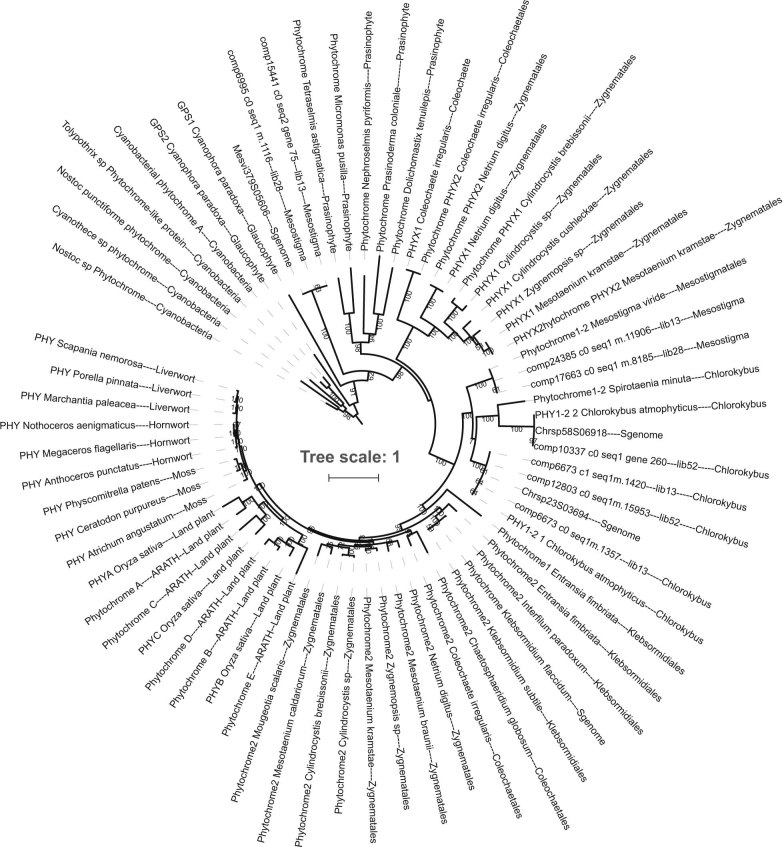
Phylogenetic tree of phytochrome. Maximum Likelihood was used to infer the phylogenetic tree of the phytochrome. The tree derived from a MAFFT alignment and constructed using RAxML (see Methods). Bootstrap values (500 replicates) ≥50% are shown.

The origin of the canonical embryophyte phytochrome (p-PHY) can be traced to the ancestor of extant streptophyte algae^37^. Two phytochrome genes were identified in both the *M. viride* and *C. atmophyticus* genome, which is in accordance with a previous transcriptome study^37^ (Extended Data Fig. 5). Interestingly, the two genes of *M. viride* showed differing domain structures, one gene containing a response regulator at the C terminus regulatory module (REC), which was not identified in the previous transcriptome study^37^. Phylogenetic analyses indicated that a gene duplication occurred in the ancestor of streptophytes, followed by loss of REC in one of the duplicated genes, resulting in the evolution of p-PHY (Fig. 5b). Non-canonical phytochromes (PHYX1 and PHYX2) were retained in several streptophyte algal lineages.

## Discussion

The successful colonization of the terrestrial landscape by plants, and their subsequent rapid evolution, is considered to be a pivotal event in the evolution of life. Here, we present the draft genomes of two early-diverging streptophyte algae that thrive in contrasting habitats: *M. viride* is found in the benthos of small shallow ponds, whereas *C. atmophyticus* is a subaerial/terrestrial alga. All previous phylogenomic analyses, including the present study, placed the two species as sister taxa in the earliest-diverging clade of streptophytes^14–17^. Their contrasting habitats and phylogenetic position make the genomes of *M. viride* and *C. atmophyticus* an exciting resource for comparative investigations into land plant evolution. Our genome analyses suggest that early-diverging streptophyte algae already took the first step on the long road to plant terrestrialization and harbour many embryophyte-type genes. Compared to Chlorophyta, the main gene family gains and expansions in early-diverging streptophyte algae were in plant hormone signal transduction, plant–pathogen interactions and TFs and regulators, most related to environmental stresses (light, temperature, salt, drought and pathogens) that are thought to have played a major role in plant terrestrialization.

*Mesostigma viride* and *C. atmophyticus* differ distinctively in their life history and cellular organization. To test whether these differences between the two species are responsible for their genome differences, we analysed the complement of flagellar genes. We detected 204 of 397 *Chlamydomonas* flagellar proteins in the *M. viride* genome. Similar numbers of conserved flagellar proteins exist in streptophyte algae that produce reproductive flagellate cells (zoospores, spermatozoids) during their life history (240 in *K. nitens* and 209 in *C. braunii*), suggesting that the ancestral number of flagellar proteins in Streptophyta was ~200–250. Second, we looked for possible differences in their life histories focusing on sexual reproduction (in *C. atmophyticus* sexual reproduction is unknown, in *M. viride* only a preliminary report exists). The core-set of meiosis-specific genes (10) were present in *C. atmophyticus*, whereas the *M. viride* genome lacked three of them. This suggested that both species reproduce sexually, although the process itself remains poorly understood.

Further insight into the differences in gene composition between *M. viride* and *C. atmophyticus* was obtained by focusing on genes involved in adaptations to the different habitats in which both species thrive. With the streptophyte algal phylogeny basically resolved, we mapped TF/TR genes on the phylogenetic tree and determined putative gains, losses, expansions and contractions of TF/TR gene family members. From this, we conclude that TFs/TRs that regulate growth and development in responses to light, salt and pathogen stresses in embryophytes originated in the common ancestor of streptophytes. Interestingly, some of the TFs/TRs thought to be involved in responses to light, salt and pathogen stresses, such as the HD-ZIPs_I–II, SRS and GRF, were absent in *M. viride*, perhaps lost in relation to its aquatic habitat. This hypothesis is consistent with the previous genome analysis of *C. braunii* (a structurally complex, streptophyte alga from an aquatic, benthic habitat) that also seems to have lost an HD-ZIP gene (HD-ZIP_IV), another HD-gene (HD_KNOX 1) and GRF^7^; HD-ZIP genes have also been lost in some secondarily aquatic embryophytes^38^.

Although elements of several biosynthetic and signalling phytohormone pathways were identified in *M. viride* and *C. atmophyticus*, they were mostly incomplete and only the CK pathway, considered to be involved in abiotic stress responses^39^, was fully recovered. Ethylene-related signalling homologues have previously been reported in *Spirogyra pratensis* and *Coleochaete orbicularis*^40,41^, and this is consistent with our findings. *K. nitens* also displays most of the ethylene-related genes, including the TF EIN3/EIL which is lacking in *M. viride* and *C. atmophyticus*, suggesting that this TF had its origin in the common ancestor of *K. nitens* and derived streptophytes^6^. The ABA pathway was also nearly complete but lacked the receptor PYL. A PYL orthologue was recently identified in a transcriptomic analysis of the streptophyte alga *Zygnema*^9^ (Zygnematophyceae). The absence of the F-box-mediated AUXIN, JA, GA and SL signalling pathways in the early-diverging streptophyte algae is not surprising, as previous studies have reported orthologues of most of the signalling components of these pathways in genomes of embryophytes but not of algae^5–7,42,43^. Interestingly, we identified the origin of several signalling components involved in phytohormone pathways. The PIN protein, an important auxin efflux carrier^44^, seems to have originated in the common ancestor of streptophytes (two PINs were detected in *M. viride*) or even in the common ancestor of Viridiplantae (contrary to a previous study, we detected the presence of PIN also in the Chlorellaceae^45^ (Chlorophyta)).

Previous studies have shown that the most important core cell wall polysaccharides of embryophytes, namely cellulose, mannan, xyloglucan, xylan and pectin, are also represented in diverse lineages of streptophyte algae^12^. The cellulose synthase-like gene families are among the most important players involved in the formation of plant cell walls^46^. Three CESA/CSLD-like homologues were identified in the *C. atmophyticus* genome, which showed high similarity to the respective genes in embryophytes. Our analyses also revealed the presence of putative CSL enzymes (CSLA/CSLC-like) in both genomes, but no CESA in *M. viride*, the latter corroborating a previous analysis using expressed sequence tags^47^. Our phylogenetic analysis indicated that the CESAs and CSLDs of embryophytes were derived from CESA/CSLD-like homologues present in the common ancestor of streptophytes that were lost in *M. viride*.

Moreover, homologues of biosynthetic genes for Rhamnogalacturonan-I and -II (RG), which are considered among the evolutionarily youngest cell wall polysaccharides of embryophytes^12^, such as RGXT and GALSs, were also identified in *C. atmophyticus* but not in *M. viride*. We also found cellulases in both *C. atmophyticus* and *K*. *nitens* but not in *M. viride*, indicating that *M. viride* lost these enzymes together with cellulose when adapting to a flagellate life history and an aquatic habitat. It is likely that mannan performs the function of cellulose in *M. viride* and may be restricted to a putative zygotic stage. Xyloglucan and xylan presumably evolved later (in either the common ancestor of *K. nitens* and other streptophytes or in the ancestor of derived streptophyte algae^12,48,49^). In summary, *M. viride* and *C. atmophyticus* differ considerably in their CAZymes, as expected from variation in their cellular organization, life history and habitats, but also from later-diverging streptophyte algae such as *K. nitens*.

Our analyses of phytochromes in the genomes of *M. viride* and *C. atmophyticus* corroborate previous transcriptome studies^37^, in that the origin of the canonical embryophyte phytochrome (p-PHY) can be traced to the common ancestor of streptophytes. We tentatively identified a gene duplication event in the common ancestor of streptophytes, followed by loss of the response regulator (REC) and the histidine phosphorylation site (H) in one of the duplicated genes. Notably, both phytochrome genes of *C. atmophyticus* clustered with canonical embryophyte phytochromes (p-PHY) as sisters of p-PHY of *M. viride*.

## Conclusions

Two major conclusions can be drawn from the comparative analysis of the draft genomes of *M. viride* and *C. atmophyticus*: first, the common ancestor of *M. viride* and *C. atmophyticus* had already developed traits that reflect adaptations to a subaerial/terrestrial habitat, exemplified by the presence of the canonical embryophyte photoreceptor phytochrome (p-PHY), evolution of TFs implicated in responses to various abiotic and biotic stresses, near-complete pathways for several phytohormones involved in stress signalling and evolution of orthologues of cellulose synthase and cellulose synthase-like genes characteristic of embryophytes. The common ancestor of streptophytes had thus taken the first step toward plant terrestrialization, supporting a recent hypothesis that streptophyte algae lived on land before the emergence of embryophytes^50^.

Second, the genomes of *M. viride* and *C. atmophyticus* differ conspicuously in genome size, structure and gene complement, revealing the dynamic nature of their genomes perhaps in response to adaptations to their contrasting habitats. Furthermore, the phylogenetic relationship of *M. viride* and *C. atmophyticus* as sister taxa leads to the conclusion that *M. viride* lost several traits of a subaerial/terrestrial ancestry following transition to a benthic, freshwater habitat, a situation that finds a parallel in the secondarily aquatic *C. braunii*^7^. Although adaptations to terrestrial life progressed stepwise in streptophyte algae, this did not follow a linear path with deviations into aquatic habitats apparently occurring repeatedly, requiring careful comparative genomic and phylogenomic analyses of a larger taxon set of streptophyte algae in future studies.

## Methods

### Culture, nucleic acid extraction and light microscopy

Axenic cultures of *M. viride* (CCAC 1140) and *C. atmophyticus* (CCAC 0220) were obtained from the Culture Collection of Algae at the University of Cologne, and grown in Waris-H culture medium^51^ (http://www.ccac.uni-koeln.de/). During all steps of culture scale-up until nucleic acid extraction, axenicity was monitored by both sterility tests and light microscopy. Total RNA was extracted from *M. viride* using the Tri Reagent Method, and from *C. atmophyticus* using the CTAB-PVP method as described in ref. ^52^. Total DNA was extracted using a modified CTAB protocol^53^ (details below). Light microscopy was performed with a Leica DMLB light microscope using a PL-APO ×100/1.40 numerical aperture (NA) objective, an immersed condenser (NA, 1.4) and a Metz Mecablitz 32 Ct3 flash system.

### Genome sequencing, data preparation and genome assembly

Paired-end libraries with insert sizes of 170 bp, 200 bp, 500 bp, 800 bp, 2 kb, 5 kb, 10 kb and 20 kb were constructed following standard Illumina protocols. The libraries were sequenced on an Illumina HiSeq 2000/4000 and BGI-seq 500 platform. A total of 245 Gb (about 746.86X) and 66.46 Gb (about 775.68X) paired-end data were generated for *M. viride* (CCAC 1140) and *C. atmophyticus* (CCAC 0220), respectively. To reduce the effect of sequencing error on assembly, we performed a rigorous quality control of raw data. We used CLC Assembly Cell (v.5.0.1)^54^ to trim the adaptors, remove duplicates and trim low-quality bases. We then used Pairfq (v.0.16.0) (https://github.com/sestaton/Pairfq) to pair the reads. Finally, SOAPfilter (v.2.2) was used to filter the reads again. After filtering off duplicated and low-quality reads and those with adaptor sequences, 74.63 and 14.51 Gb high-quality clean reads remained for *M. viride* (CCAC1140) and *C. atmophyticus* (CCAC 0220), respectively, which were then subjected to a pipeline for genome assembly.

*k*-mer analysis was performed to survey genome size, heterozygosity and repeat content before genome assembly. The peak of *k*-mer frequency (*F*) is determined by the total reads number (*N*), genome size (*G*), read length (*L*) and the length of *k*-mer (*K*), following the formula: *F* = *N* × (*L* – *K* + 1)/*G*. Total *k*-mer number (*M*) is determined by the formula *M* = *N* × (*L* – *K* + 1). As a result, genome size can be calculated by *G* = *M*/*F*. This formula enables accurate estimation of *G*, and hence an estimation of genome size for homozygous diploid or haploid genomes. All the above analyses indicated homozygosity of the genome and gave similar estimations of genome size. The final genome size estimate (329 Mb for *M. viride* and 85.68 Mb for *C. atmophyticus*) was obtained through 17-mer analysis.

We carried out the SPAdes (v.3.10.1)^55^ genome assembly algorithm to assemble the contigs of *M. viride*. The contigs were formed without gaps. Subsequently platanus (v.1.2.4) was conducted to construct the scaffolds, from short insert-sized paired-end reads to long insert-sized paired-end reads. To extend the assembly and close gaps, the scaffolder SSPACE (v.3.0)^56^ was applied to extend the scaffolds while GapCloser (v.1.12) was used to close gaps and extend the scaffolds again. For *C. atmophyticus*, the assembly was generated by SOAPdenovo-127-mer (v.2.04)^57^ with a *k*-mer of 85. To close the gaps within the constructed scaffolds, we used paired-end reads mapped to the scaffolds using GapCloser (v.1.12). Variant detection was done using Pilon (v.2.11) to improve draft assembly quality.

The quality of the assembly was evaluated in four ways. First, we used BUSCO (v.3)^58^ to determine the proportion of a core-set of 303 highly conserved eukaryotic genes present in the genomes of *M. viride* and *C. atmophyticus*. Second, Soap (v.2.21) was used to map the reads to the draft assemblies to evaluate the DNA reads mapping rate in both species. Meanwhile, sequence depth and genetic copy content distribution were calculated. Third, we used BLAT (v.36)^59^ to compare the draft assemblies to a transcript assembled by Bridger. Finally, we mapped the RNA reads to the draft assemblies to evaluate the RNA reads mapping rate using Tophat2 (ref. ^60^).

### Transcriptome sequencing and analysis

For Illumina sequencing, we considered two ways of library construction. The ribosomal RNA-depleted RNA library was constructed using the ribo-zero rRNA removal kit (plant) (Illumina) following the manufacturer’s protocol, while the poly(A)-selected RNA library was constructed using the ScriptSeq Library Prep kit (Plant leaf) (Illumina) following the manufacturer’s protocol. These libraries were sequenced on the Illumina sequencing system. All of the sequenced data were then assembled into transcripts following the Bridger pipeline. This set of transcript sequences was used for assessing the accuracy of the genome assembly and for gene annotation.

Gene expression was measured as fragments per kilobase of transcript per million mapped reads.

### Detection and classification of repetitive elements

Three types of repeat (DNA transposon elements, retrotransposon elements and tandem repeats) were identified in the genomes of *M. viride* and *C. atmophyticus*. DNA transposons and retrotransposon elements were identified using MITE-hunter^61^ and LTRharvest^62^, respectively. RepeatModeler (v.1.0.8) was used to search for other repeats using a de novo approach. Alternatively, RepeatMasker^63^ was applied to using a custom library comprising a combination of Repbase and a de novo-predicted repetitive element library.

### Gene prediction

We used a combination of de novo gene prediction methods, homology-based search methods and RNA sequencing-aided annotation methods. For de novo gene prediction, PASApipeline v.2.1.0 was applied to predict gene structure using transcripts assembled by Bridger, after which the inferred gene structures were used in AUGUSTUS (v.3.2.3)^64^ to train gene models based on transcript evidence. In addition, GeneMark (v.1.0)^65^ was used to build a hidden Markov model based on genome sequence. For homology-based annotation, we selected gene sets from certain model green algae. In regard to RNA sequencing-aided methods, we took the transcripts assembled by Bridger as evidence. The final consensus gene sets were generated by combining all the evidence using MAKER (v.2.31.8)^66^. The result of the first round was used for SNAP to train another hidden Markov model based on transcriptome. Subsequently, the hidden Markov model was added to MAKER.

The final gene set was evaluated with two approaches. The BUSCO core eukaryotic gene-mapping approach was used to determine gene set completeness; RNA read mapping was another means of evaluation. We mapped the RNA reads to the gene set with Tophat2, while coverage depth was calculated by Samtools (v.0.1.19).

Gene function annotation was performed by BLASTP (1 × 10^–5^) against several known databases, including SwissProt, TrEMBL, Kyoto Encyclopedia of Genes and Genomes (KEGG), COG and NR. InterProScan (using data from Pfam, PRINTS, SMART, ProDom and PROSITE) was used to identify protein motifs and protein domains of the predicted gene set. Gene Ontology information was obtained through Blast2go (v.2.5.0).

### Comparative genome analyses and phylogenetics

The genomes of *M. viride* and *C. atmophyticus* were compared to those of nine other algae, namely *Cyanophora paradoxa*, *Chondrus crispus*, *C. merolae*, *M. commoda*, *Ostreococcus tauri*, *Chlorella variabilis*, *Volvox carteri* and *Chlamydomonas reinhardtii*, with the Streptophyta *C. braunii, K. nitens, Physcomitrella patens, Selaginella moellendorffii, Oryza sativa* subsp. *Japonica* and *Arabidopsis thaliana*. The same species were used to define orthogroups (using OrthoFinder, v.1.1.8). Single-copy gene families (that is, gene families with only one gene member per species) were used to construct phylogenetic trees based on maximum likelihood. We first performed multiple sequence alignment by MAFFT (v.7.310) for each single-copy gene orthogroup, followed by gap position removal (only positions where 50% or more of the sequences have a gap are treated as a gap position). A maximum-likelihood phylogenetic tree was constructed for each single-copy orthogroup. Next, we used ASTRAL to combine all single-copy gene trees to a species tree with the multi-species coalescent model. The online tool iTOL was performed to edit and display the final phylogenetic tree.

### Gene identification

We used various search methods to identify different genes.

For TFs and TR we used the HMMER search method. We downloaded the HMMER model of the domain structure of each TF from the Pfam website (https://pfam.xfam.org/), referring to the TAPscan (v.2) TF database (https://plantcode.online.uni-marburg.de/tapscan/). Preliminary candidates were collected by searching the HMM profile for each species (<1 × 10^–10^). Then, we filtered genes that did not match the SwissProt functional annotation (<1 × 10^–5^). Finally, we filtered genes containing an incorrect domain according to the domain rules of the TAPscan (v.2) TF database. Most TFs/TRs were confirmed by phylogenetic analysis.

The HMMER search method was also used for phytohormone signalling pathways. We collected ~10–20 query genes from representative model organisms (for example, *A. thaliana* and *C. reinhardtii*). A custom profile HMM was built (hmmer-3.1b2) based on the query genes for each phytohormone. All signalling pathway genes must pass the following two restrictions: (1) genes should pass the HMM profile built by ~10–20 known genes (<1 × 10^–10^), and (2) genes should match the SwissProt functional annotation (<1 × 10^–5^). The custom profile HMM is available in the Supplementary Information (HMM profiles).

The genes involved in other pathways, such as biosynthetic phytohormone pathways and cell wall-related genes, were selected based on two criteria : (1) BLAST by known query genes (<1 × 10^–5^), and (2) matching based on SwissProt functional annotation (<1 × 10^–5^).

For CAZyme annotation we used the dbCAN2 metaserver (http://bcb.unl.edu/dbCAN2/index.php). This server integrates three tools/databases for automated CAZyme annotation: (1) HMMER for annotation of the CAZyme domain against the dbCAN CAZyme domain HMM database; (2) DIAMOND for fast BLAST hits in the CAZy database; and (3) Hotpep for short conserved motifs in the PPR library.

We also constructed phylogenetic trees to classify certain highly similar genes, including ARF, CSL, HD-ZIP, YUCCA, PIF, TGA and others (see Supplementary Figs. 6–13). For phylogenetic analysis of individual genes, we first performed multiple sequence alignment by MAFFT (v.7.310) for each single-copy gene orthogroup, followed by removal of gap position (only positions where 50% or more of the sequences have a gap are treated as gap positions). Then, a maximum-likelihood phylogenetic tree was constructed by RAxML (amino acid substitution model: CAT + GTR, with 500 bootstrap replicates).

### Analysis of significantly increased/decreased gene numbers of gene families

We used a median gene number to estimate the changes in gene family size^6^, shown in Fig. 1d. These showed the gene families whose numbers of genes were significantly increased in embryophytes compared to algae, by calculating the median of the embryophyte gene number/median of the algal gene number ≥10. Taking Rhodophyta and Streptophyta as an example, we selected five representative species for each lineage. Gene numbers of these lineages in the gene family were sorted from largest to smallest. If the median of the Streptophyta gene number/median of the Rhodophyta gene number was >5, this gene family was considered a significantly increased gene family in Streptophyta. The same method is used to define the embryophytes and algal genes in Fig. 1e. If the median gene number of both embryophytes and algae was >0, we defined the genes in this family as those commonly shared between embryophytes and algae. If the median embryophyte gene number was >0 and the median algae gene number was 0, we defined the genes in this family as embryophyte genes. In special cases, if both the median embryophyte and median algae gene number was 0, we removed those low-frequency gene families.

### Conserved motif identification

The local multiple Em (expectation maximization) for motif elicitation (MEME, http://meme-suite.org/) tool was used to identify conserved motifs. All genes in this study were analysed using the classical model. According to *e* values, the number of motifs that MEME should find was set to 20.

### Reporting Summary

Further information on research design is available in the Nature Research Reporting Summary linked to this article.

## Supplementary information

Supplementary Information Supplementary Figs. 1–15.

Reporting Summary

Supplementary Table 1. Supplementary ST 1–37.

Supplementary Data 1 The HMMER profile of phytohormone genes.

Supplementary Data 2 The HMMER profile of transcription factors and transcription regulators.

## Data Availability

The whole-genome assemblies and transcriptome for *M. viride* and *C. atmophyticus* in this study are deposited at DDBJ/ENA/GenBank under accession nos. RHPH00000000 and RHPI00000000. Those data are also available in the CNGB Nucleotide Sequence Archive (accession no. CNP0000228).
