# Supplementary material for: Genomes of early-diverging streptophyte algae shed light on plant terrestrialization
Source: Nat Plants. 2019 Dec 16;6(2):95–106. doi: 10.1038/s41477-019-0560-3 (PMC7027972; doi:10.1038/s41477-019-0560-3)
Supplement: Supplementary file 1 — Supplementary Figs. 1–15. [file 41477_2019_560_MOESM1_ESM.pdf]

nature plants

SUPPLEMENTARY INFORMATION

https://doi.org/10.1038/s41477-019-0560-3

In the format provided by the authors and unedited.

Genomes of early-diverging streptophyte algae shed light on plant terrestrialization

Sibo Wang[1,2,3,12], Linzhou Li[1,4,5,12], Haoyuan Li[1,2], Sunil Kumar Sahu[1,4], Hongli Wang[1,2], Yan Xu[1,6], Wenfei Xian[1,2], Bo Song[1,2], Hongping Liang[1,6], Shifeng Cheng[1,2], Yue Chang[1,2], Yue Song[1,2], Zehra Çebi[7], Sebastian Wittek[7], Tanja Reder[7], Morten Peterson[3], Huanming Yang[1,2], Jian Wang[1,2], Barbara Melkonian[7,11], Yves Van de Peer[8,9], Xun Xu[1,2], Gane Ka-Shu Wong[1,10]*, Michael Melkonian[7,11]*, Huan Liu[1,3,4]* and Xin Liu[1,2,4]*

[1]BGI-Shenzhen, Shenzhen, China. [2]China National GeneBank, BGI-Shenzhen, Shenzhen, China. [3]Department of Biology, University of Copenhagen, Copenhagen, Denmark. [4]State Key Laboratory of Agricultural Genomics, BGI-Shenzhen, Shenzhen, China. [5]Department of Biotechnology and Biomedicine, Technical University of Denmark, Lyngby, Denmark. [6]BGI Education Center, University of Chinese Academy of Sciences, Shenzhen, China. [7]Botanical Institute, Cologne Biocenter, University of Cologne, Cologne, Germany. [8]Department of Plant Biotechnology and Bioinformatics, Ghent University and VIB/UGent Center for Plant Systems Biology, Ghent, Belgium. [9]Centre for Microbial Ecology and Genomics, Department of Biochemistry, Genetics and Microbiology, University of Pretoria, Pretoria, South Africa. [10]Department of Biological Sciences and Department of Medicine, University of Alberta, Edmonton, Alberta, Canada. [11]Present address: University of Duisburg-Essen, Campus Essen, Faculty of Biology, Essen, Germany. [12]These authors contributed equally: Sibo Wang, Linzhou Li. *e-mail: gane@ualberta.ca; michael.melkonian@uni-koeln.de; liuhuan@genomics.cn; liuxin@genomics.cn

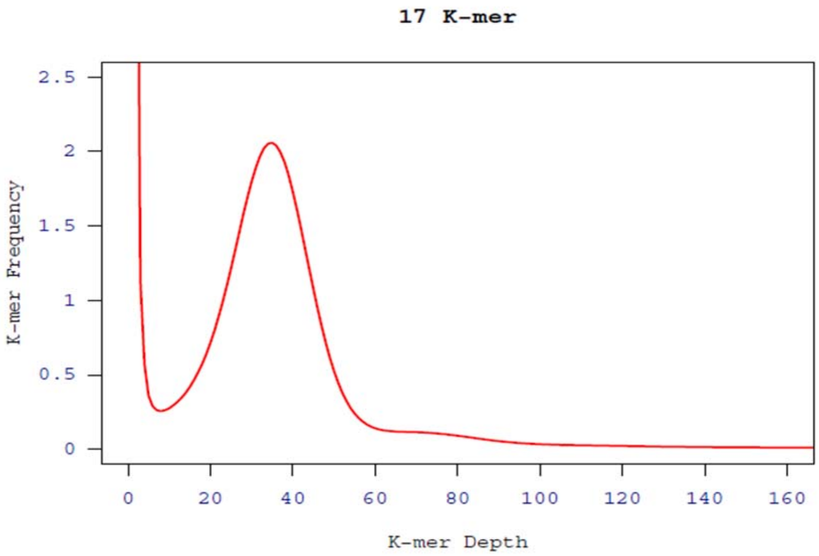

Supplementary Figure 1. 17-mer analysis to estimate genome complexity (genome size, repeat content estimation, heterozygosity calculation) of *M. viride*. The 17-mer frequency distribution generated from the sequencing reads was plotted. The peak is approximately 35 and the total K-mer count is 11,517,226,902. The genome size is estimated as about 329 Mb.

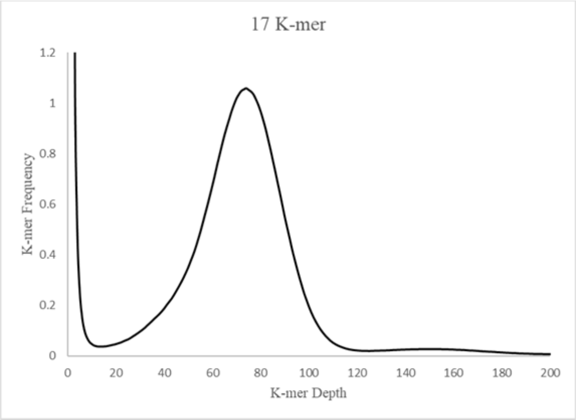

Supplementary Figure 2. 17-mer analysis to estimate genome complexity (genome size, repeat content estimation, heterozygosity calculation) of *C. atmophyticus*. The 17-mer frequency distribution generated from the sequencing reads was plotted. The peak depth is about 74 and the total K-mer count is 6,340,143,223. The genome size was estimated as about 85.68 Mb., low heterozygosity rate and repeat content can be observed.

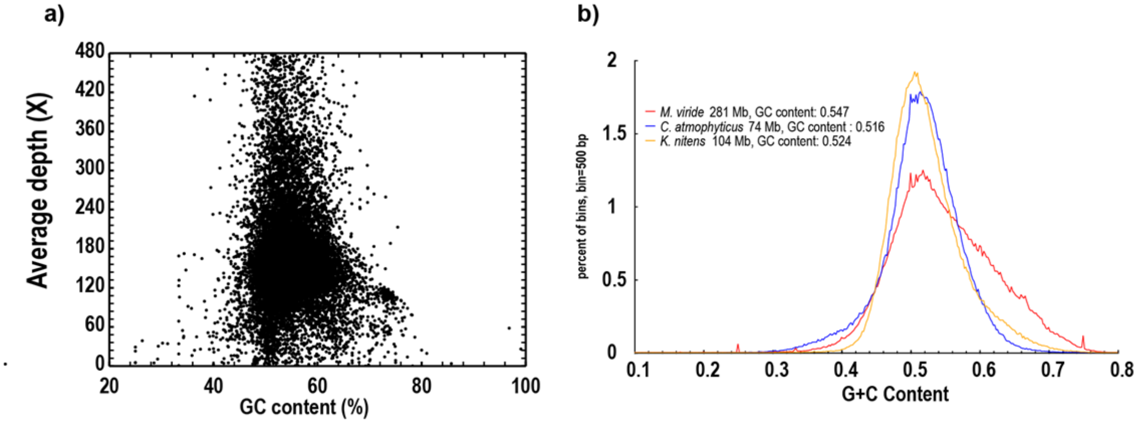

Supplementary Figure 3. Assessment of genomic data for assembly of *M. viride*. (a) The GC content and the average depth were calculated from 10 kb non-overlapping sliding windows. The distribution pattern of the GC content indicates a relative pure single genomic sample without contaminations; (b) comparison of GC content across closely related species and model species.

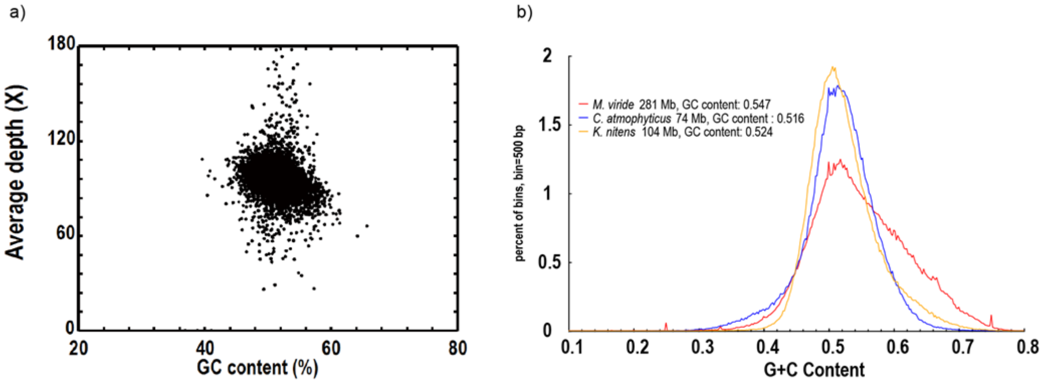

Supplementary Figure 4. Distribution of GC depth of *C. atmophyticus*. (a) The GC content and the average depth were calculated from 10 kb non-overlapping sliding windows. The distribution pattern of GC content indicates a relative pure single genomic sample without contamination and no GC bias; **(b)** comparison of GC content across closely related species;

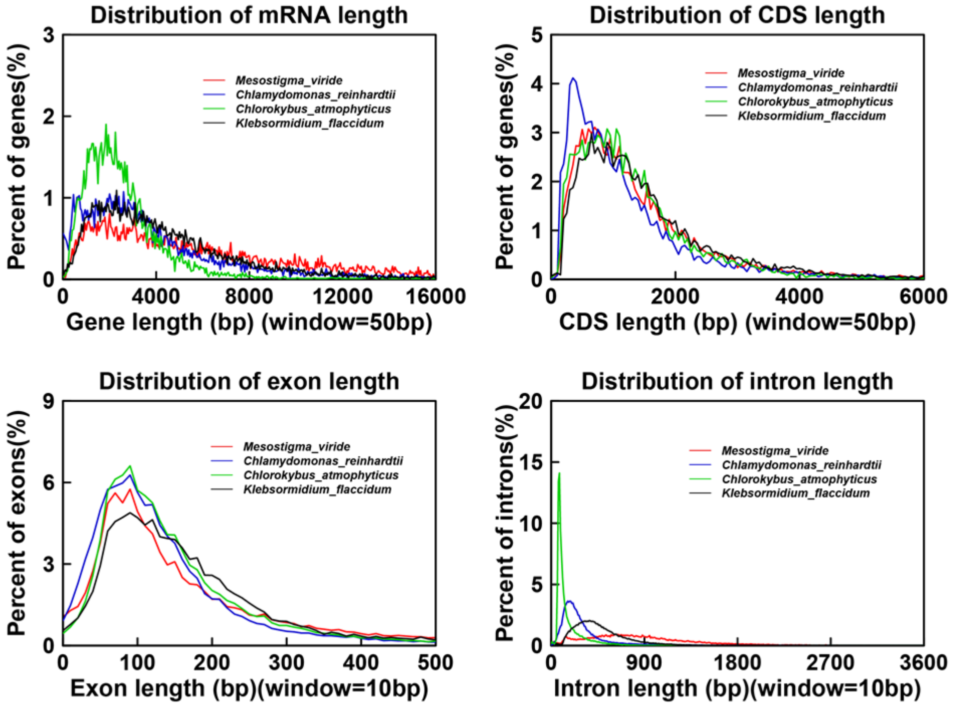

Supplementary Figure 5. Comparison of gene structural features among four green algal species. The distributions of mRNA length, CDS length, exon length and intron length for *M. viride* and *C. atmophyticus* genome were compared against other relative species.

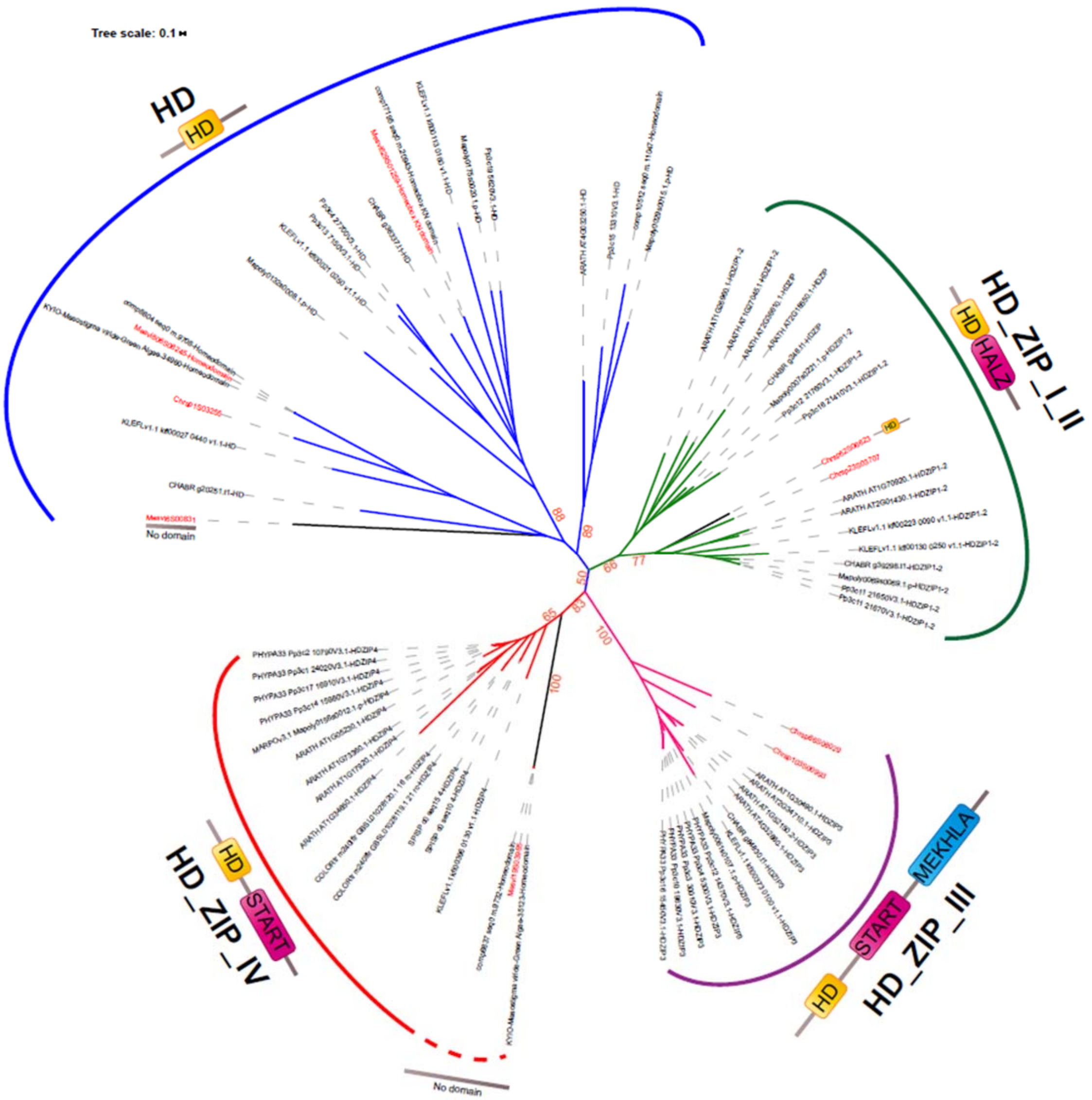

Supplementary Figure 6. Phylogenetic tree of the transcription factor HD and HD-Zip. The tree derived from a MAFFT alignment and constructed using IQ-TREE (see Methods). Bootstrap values (200 replicates) ≥50% are shown. The sequences derived from the *M. viride* and *C. atmophyticus* genomes are highlighted in red. The tree also included the sequences derived from the transcriptomes of *M. viride* and *C. atmophyticus*. Each clade was marked with the domain structure information and HD-ZIP subfamily name. Although one of *M. viride* genome sequence and its corresponding transcriptome sequence clustered with HD-ZIP IV, these sequences display no domain structure (marked as dashed red line).

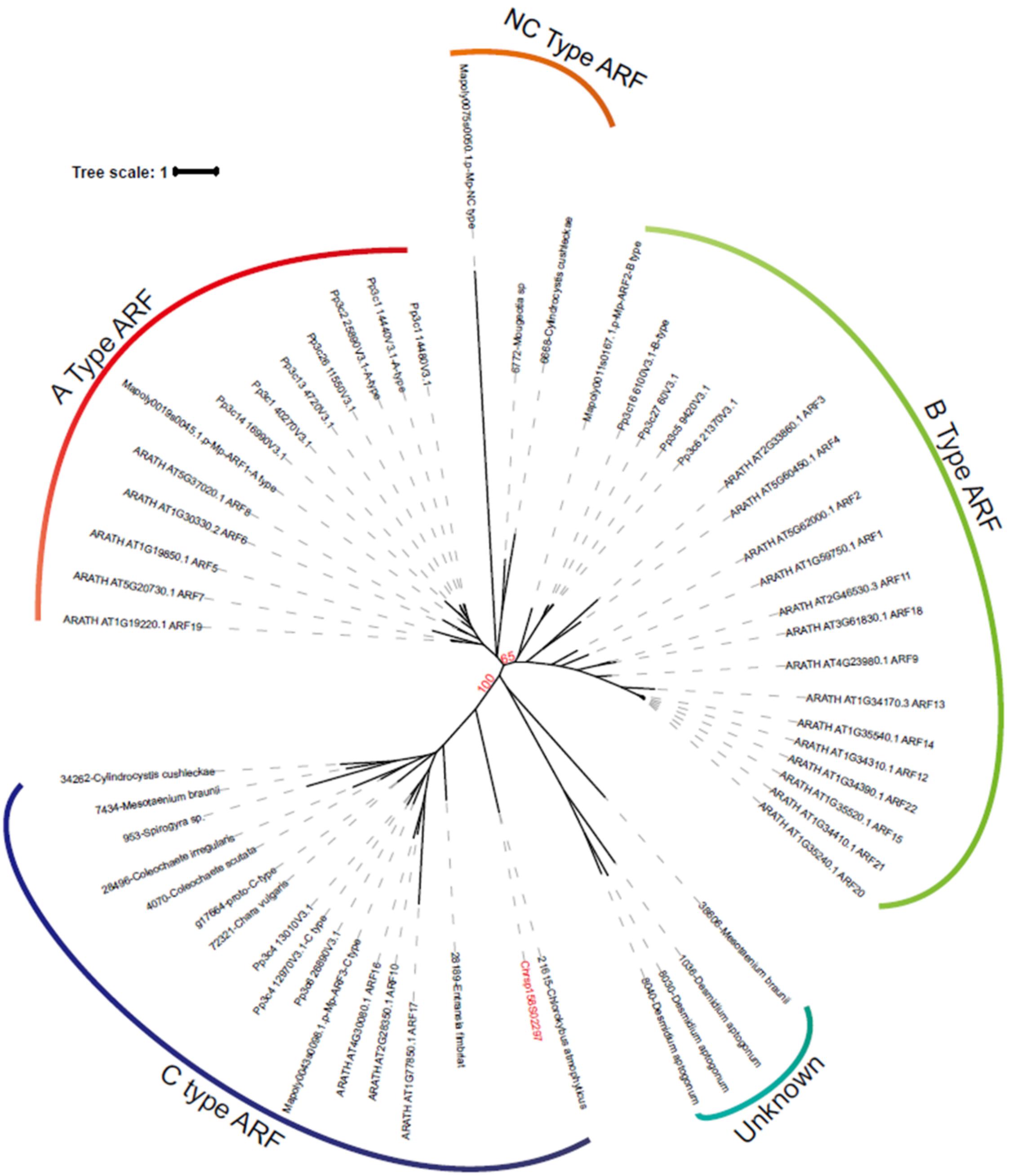

Supplementary Figure 7. Phylogenetic tree of the Auxin response factor (ARF). (a) The tree derived from a MAFFT alignment and constructed using IQ-TREE (see Methods). Bootstrap values (200 replicates) ≥50% are shown. The sequence derived from the *C. atmophyticus* genome is highlighted in red. The tree also included the sequence derived from transcriptome of *C. atmophyticus* (21615-Chlorokybus_atmophyticus). (b). Conserved motifs of each sequence were identified in the respective clade through MEME analysis. 20 motifs were identified.

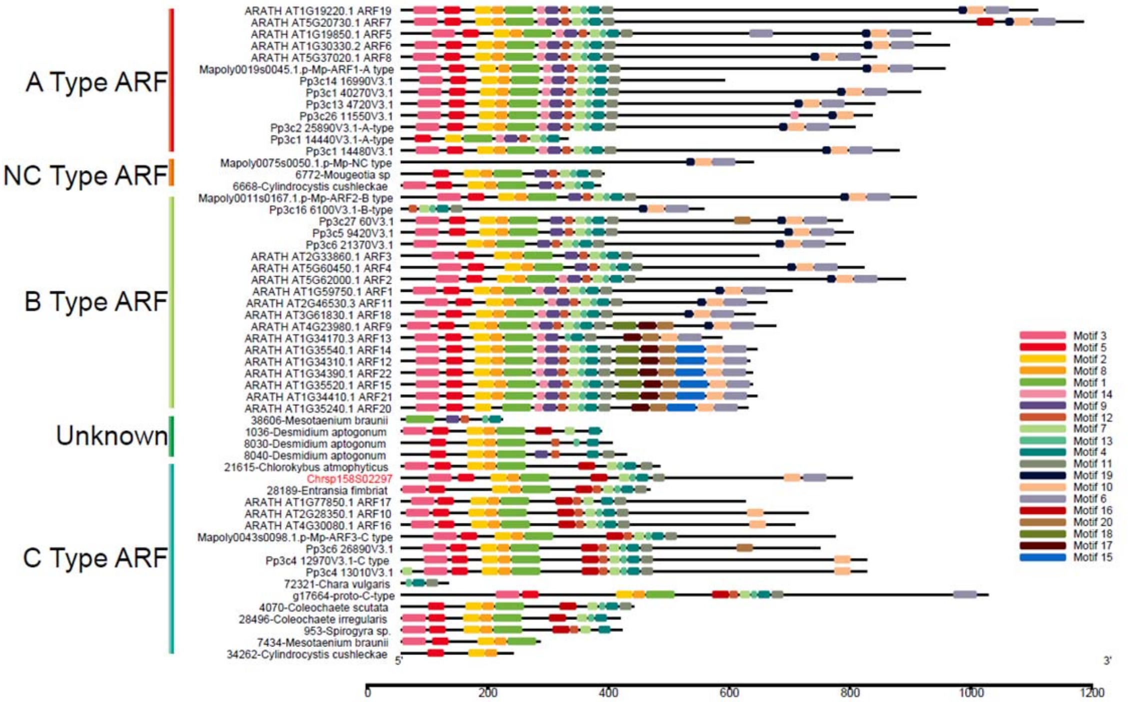

Supplementary Figure 8. Conserved motifs of each sequence were identified in the respective clade through MEME analysis of the Auxin response factor (ARF). 20 motifs were identified.

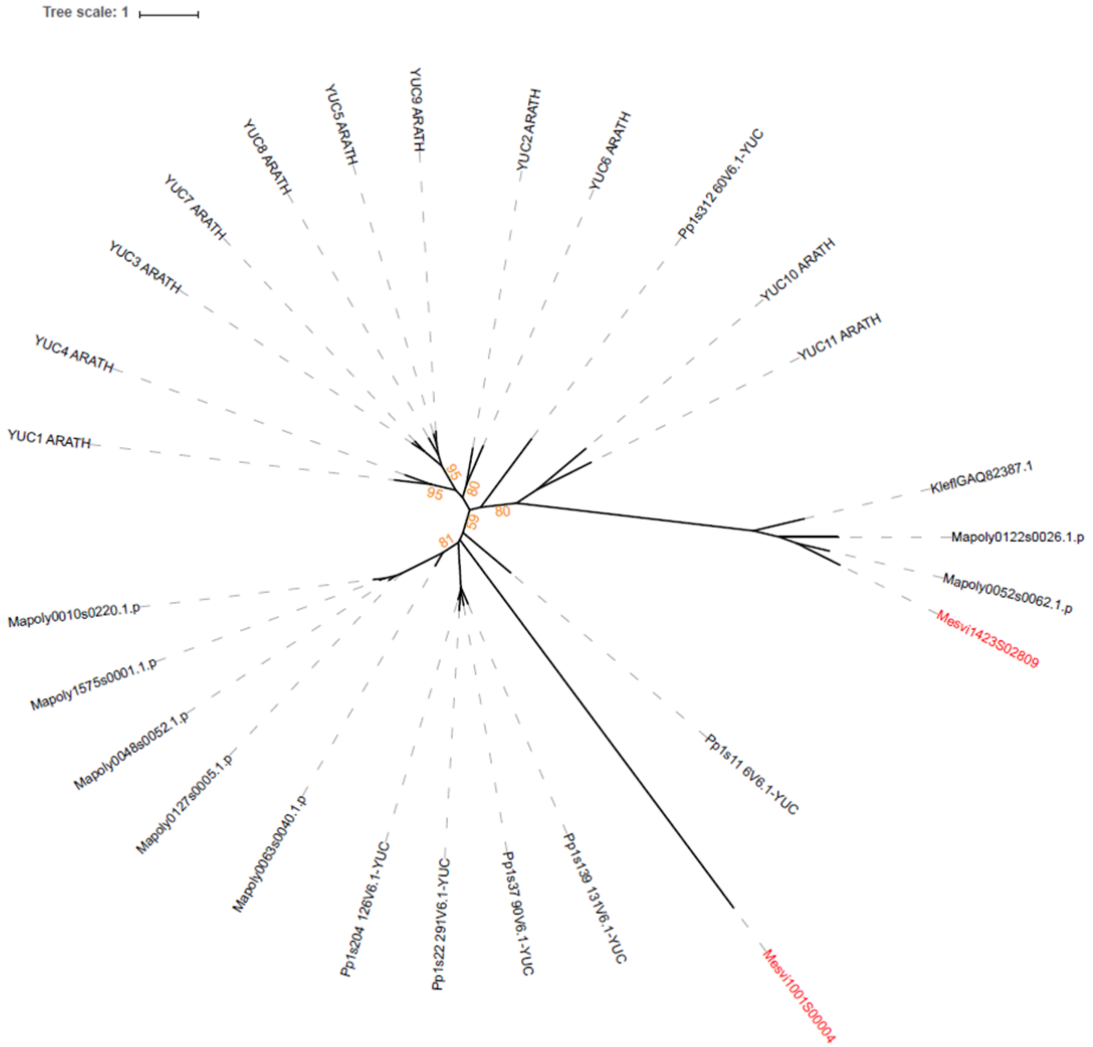

Supplementary Figure 9. Phylogenetic tree of YUCCA. The tree derived from a MAFFT alignment and constructed using IQ-TREE (see Methods). Bootstrap values (200 replicates) ≥ 50% are shown. The sequences derived from the *M. viride* genome are highlighted in red. The tree includes all identified YUCCA sequences of *Arabidopsis thaliana*, *Marchantia polymorpha*, *Physcomitrella patens* and *Klebsormidium nitens* downloaded from Uniport database (https://www.uniprot.org).

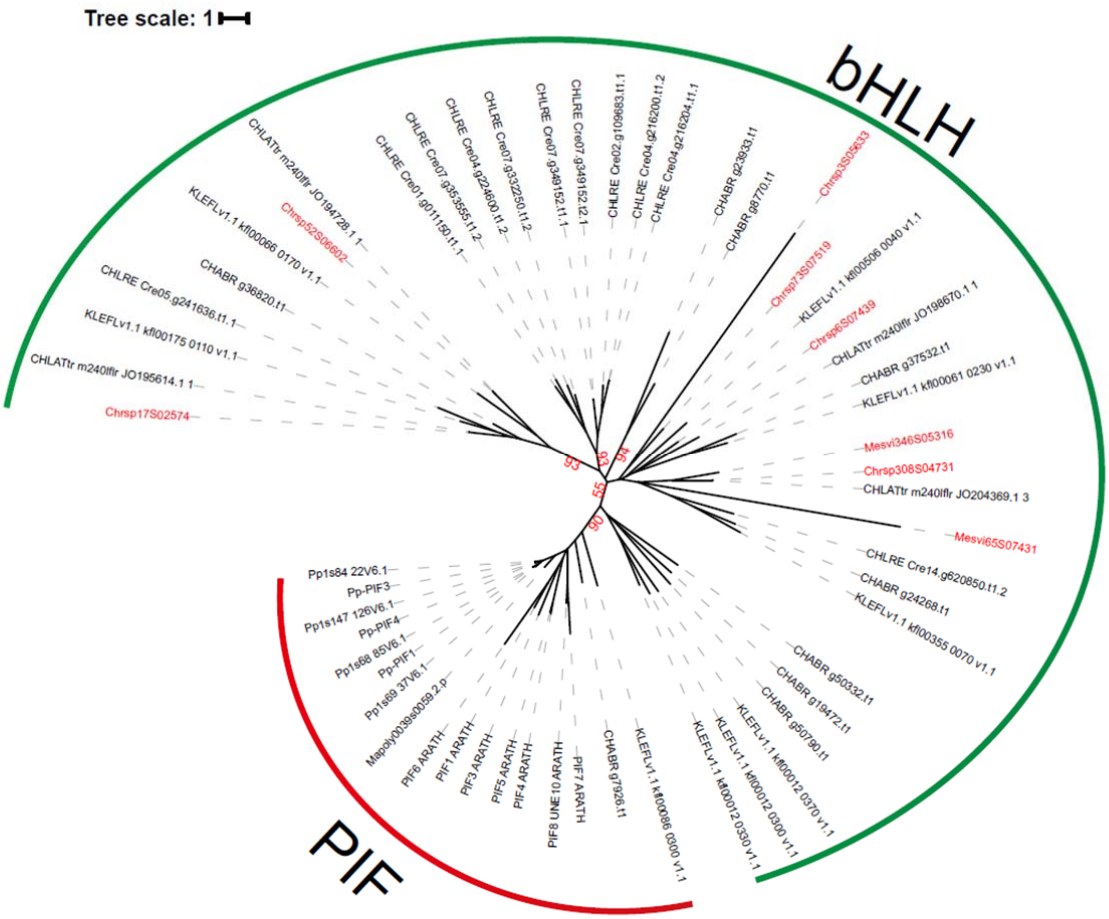

Supplementary Figure 10. Phylogenetic tree of PIF and bHLH. The tree derived from a MAFFT alignment and constructed using IQ-TREE (see Methods). Bootstrap values (200 replicates) ≥50% are shown. The sequences derived from the *M. viride* and *C. atmophyticus* genome are highlighted in red. The tree includes all the identified PIF sequences of *Arabidopsis thaliana* collected from the Uniport database (https://www.uniprot.org).

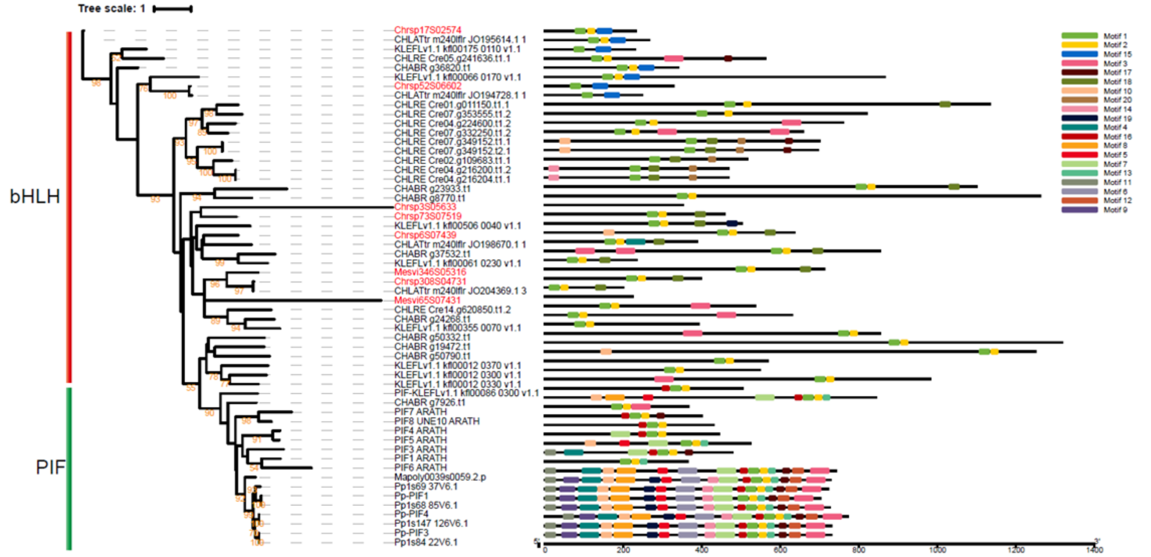

Supplementary Figure 11. Conserved motifs of each sequence were identified in the respective clade through MEME analysis of PIF and bHLH. 20 motifs were identified.

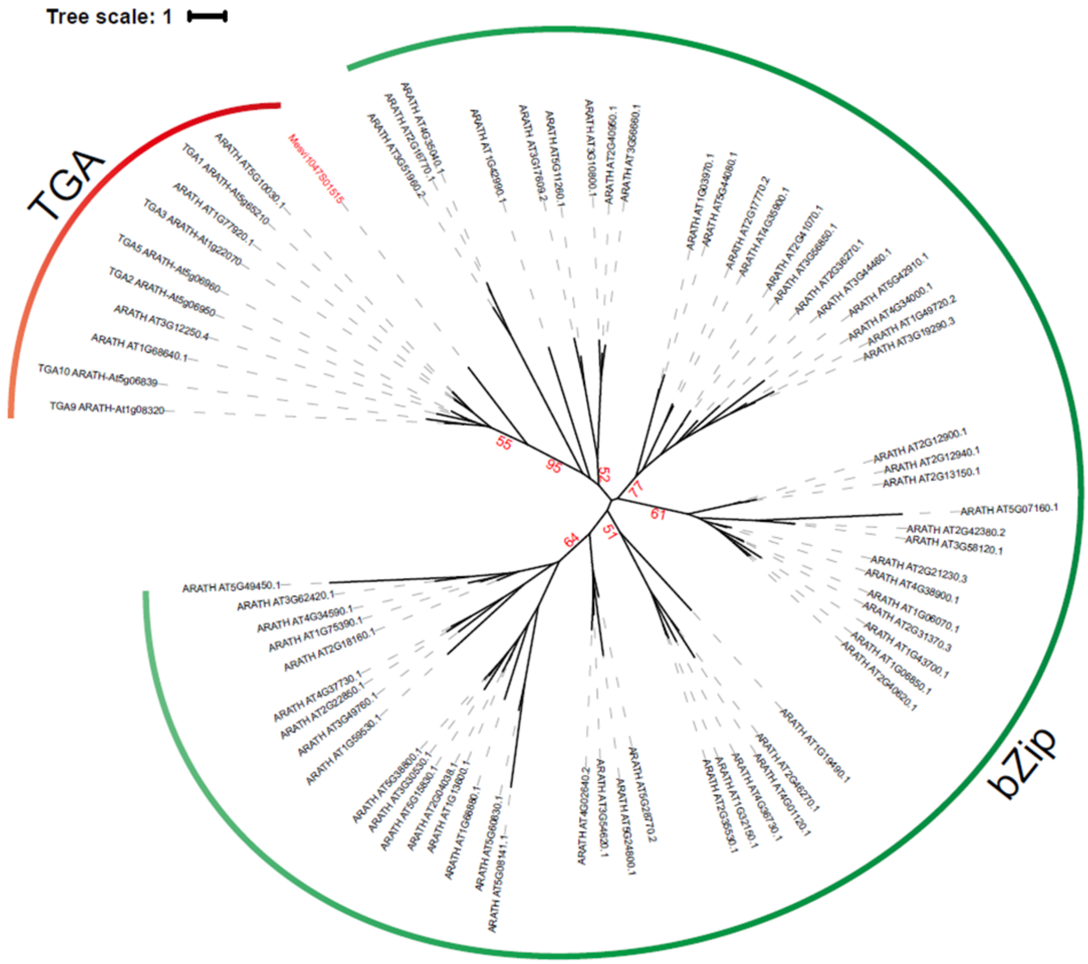

Supplementary Figure 12. Phylogenetic tree of TGA and bZIP. The tree derived from a MAFFT alignment and constructed using IQ-TREE (see Methods). Bootstrap values (200 replicates) ≥50% are shown. The sequences derived from *M. viride* and *C. atmophyticus* genome are highlighted in red. The tree includes all the identified TGA sequences of *Arabidopsis thaliana* collected from Uniport database (https://www.uniprot.org).

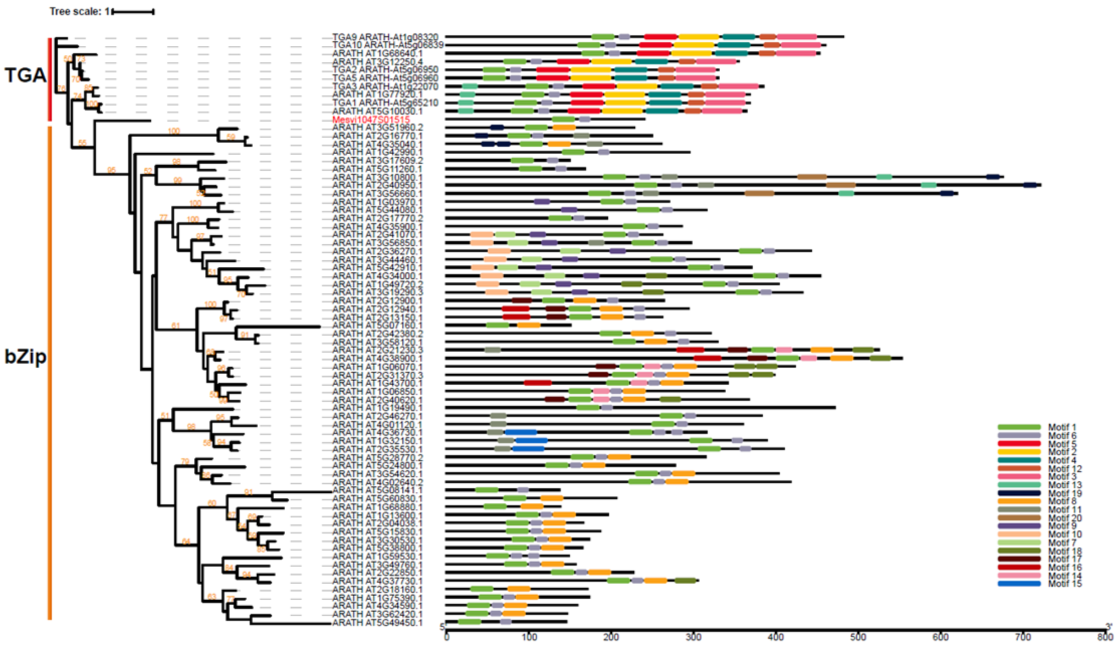

Supplementary Figure 13. Conserved motifs of each sequence were identified in the respective clade through MEME analysis of the TGA and bZIP. 20 motifs were identified.

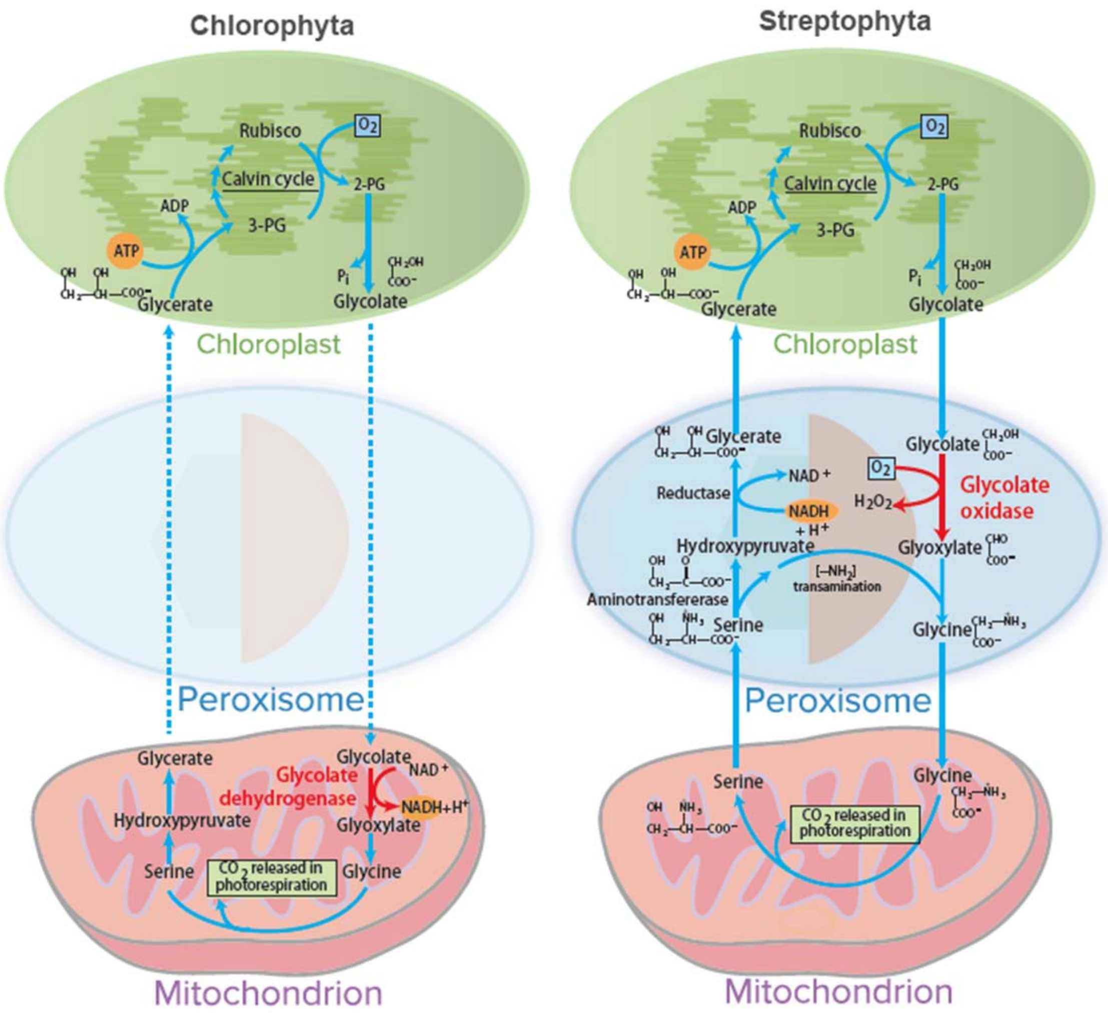

Supplementary Figure 14. The photorespiration pathway is different in Chlorophyta compared to Streptophyta. Analysis of targeting signal peptide of glycolate dehydrogenase and glycolate oxidase in Chlorophyta and Streptophyte algae. Glycolate oxidase of streptophyte algae is located in peroxisomes, but no peroxisome targeting signal was identified in the glycolate dehydrogenase of Chlorophyta. All glycolate dehydrogenases derived from Chlorophyta are located in mitochondria. However, glycolate dehydrogenase of streptophyte algae was found to be targeted to various organelles (chloroplast, mitochondria) except the peroxisomes (Table S). The major metabolites produced/involved during photorespiration in chlorophytes and streptophytes are also depicted for streptophytes, and the major differences among them are highlighted in red color.

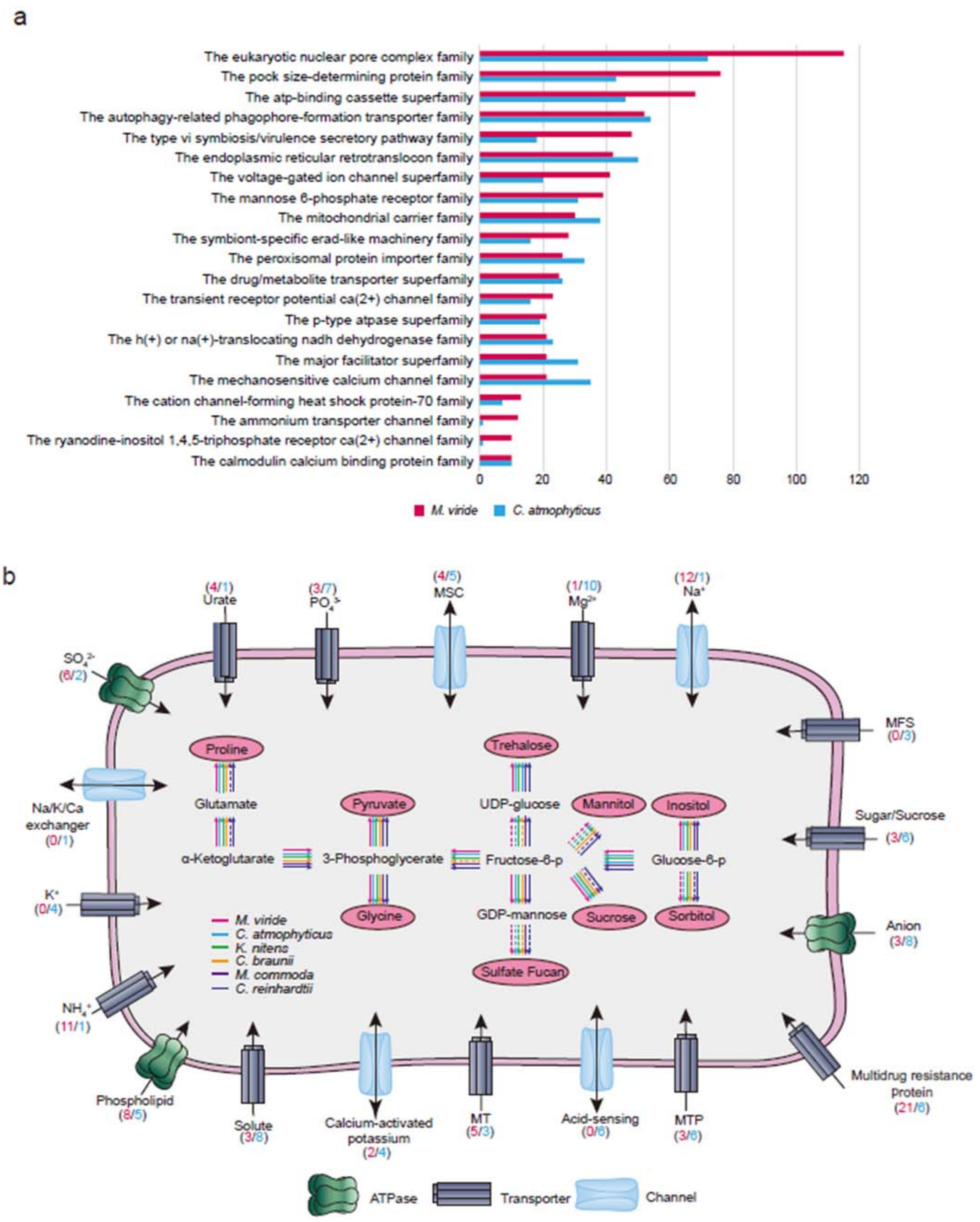

Supplementary Figure 15. Overview of the key components involved in the organic and ionic osmoregulation (a) Comparative analysis of metabolite transporters. (b) Schematic representation of the main membrane transport systems which might be involved in osmoregulation. Only the significantly different transporters, ATPase, and channels are presented in the figure. The compatible osmolyte biosynthetic pathways between representative Streptophyta and Chlorophyta are shown. Dash arrows mean incomplete pathway, while solid arrows mean complete pathway. The copy number shows in pink and blue color for *Mesostigma* and *Chlorokybus*, respectively.
