# Supplementary material for: Genomes of early-diverging streptophyte algae shed light on plant terrestrialization
Source: Nat Plants. 2019 Dec 16;6(2):95–106. doi: 10.1038/s41477-019-0560-3 (PMC7027972; doi:10.1038/s41477-019-0560-3)
Supplement: Supplementary file 2 — Reporting Summary [file 41477_2019_560_MOESM2_ESM.pdf]

nature research

Corresponding author(s): Gane Ka-Shu Wong, Michael Melkonian, Huan Liu and Xin Liu

Last updated by author(s): 14th Oct, 2019

Reporting Summary

Nature Research wishes to improve the reproducibility of the work that we publish. This form provides structure for consistency and transparency in reporting. For further information on Nature Research policies, see Authors & Referees and the Editorial Policy Checklist.

Statistics

For all statistical analyses, confirm that the following items are present in the figure legend, table legend, main text, or Methods section.

n/a	Confirmed	
☒	☐	The exact sample size (*n*) for each experimental group/condition, given as a discrete number and unit of measurement
☒	☐	A statement on whether measurements were taken from distinct samples or whether the same sample was measured repeatedly
☒	☐	The statistical test(s) used AND whether they are one- or two-sided<br>*Only common tests should be described solely by name; describe more complex techniques in the Methods section.*
☒	☐	A description of all covariates tested
☒	☐	A description of any assumptions or corrections, such as tests of normality and adjustment for multiple comparisons
☒	☐	A full description of the statistical parameters including central tendency (e.g. means) or other basic estimates (e.g. regression coefficient) AND variation (e.g. standard deviation) or associated estimates of uncertainty (e.g. confidence intervals)
☐	☒	For null hypothesis testing, the test statistic (e.g. *F*, *t*, *r*) with confidence intervals, effect sizes, degrees of freedom and *P* value noted<br>*Give P values as exact values whenever suitable.*
☒	☐	For Bayesian analysis, information on the choice of priors and Markov chain Monte Carlo settings
☒	☐	For hierarchical and complex designs, identification of the appropriate level for tests and full reporting of outcomes
☒	☐	Estimates of effect sizes (e.g. Cohen's *d*, Pearson's *r*), indicating how they were calculated

Our web collection on statistics for biologists contains articles on many of the points above.

Software and code

Policy information about availability of computer code

Data collection	Paired-end libraries with insert sizes of 170 bp, 250 bp, 500 bp, 800 bp, 2 kb, 5 kb, 10 kb and 20 kb were constructed following standard Illumina protocols. The libraries were sequenced on an Illumina HiSeq 2000/4000 and BGI-seq 500 platform. A total of 245Gb (about 746.86X) and 66.46Gb (about 775.68X) paired-end data were generated for M. viride (CCAC 1140) and C. atmophyticus (CCAC 0220), respectively.<br><br>For Illumina sequencing, we considered two ways of library construction. The rRNA-depleted RNA library was constructed using the ribo-zero rRNA removal kit (plant) (Illumina, American) following the manufacturer's protocol, while the poly (A)-selected RNA library was constructed using the ScriptSeq Library Prep kit (Plant leaf) (Illumina, American) following the manufacturer's protocol.
Data analysis	The list of Software used in this study are as follows:<br><br>CLC Assembly Cell (version 5.0.1)<br>Pairfq (version 0.16.0)<br>SOAPfilter (version 2.2)<br>Kmerfreq (version 1.0)<br>SPAdes (version 3.10.1)<br>Platanus (version 1.2.4)<br>SOAPdenovo-127mer (version 2.04)<br>SSPACE (version 3.0)<br>GapCloser (version 1.12)<br>Pilon (version 2.11)<br>BUSCO (version3)<br>Soap (version 2.21)<br>blat (v36)<br>Bridger_r2014-12-01

Trinityrnaseq (version 2.1.1)
Tophat2 (version 2.1.0)
RepeatModeler (version 1.0.8)
GenomeTools (version 1.5.8)
MITE-hunter (version 8/19/2010)
LTRharvest (version 1.0)
PASApipeline-2.1.0
AUGUSTUS (version 3.2.3)
GeneMark (version 1.0)
MAKER (version 2.31.8)
SNAP (version 2006-07-28)
Samtools (version 0.1.19)
blast-2.2.26
ncbi-blast-2.2.31+
Blast2go (version 2.5.0).
InterProScan 5.28-67.0
OrthoFinder (version 1.1.8)
hmmer-3.1b2
MAFFT (version 7.310)
RAxML (version 8.2.4)
IQ-tree (version 1.6.1)
ASTRAL (version 4.11.1)

For manuscripts utilizing custom algorithms or software that are central to the research but not yet described in published literature, software must be made available to editors/reviewers. We strongly encourage code deposition in a community repository (e.g. GitHub). See the Nature Research guidelines for submitting code & software for further information.

Data

Policy information about availability of data

All manuscripts must include a data availability statement. This statement should provide the following information, where applicable:

- Accession codes, unique identifiers, or web links for publicly available datasets
- A list of figures that have associated raw data
- A description of any restrictions on data availability

The whole genome assemblies for M. viride and C. atmophyticus in this study are deposited at DDBJ/ENA/GenBank under the accession numbers of RHPH00000000 and RHPI00000000. Those data are also available in the CNGB Nucleotide Sequence Archive (CNSA: http://db.cngb.org/cnsa; accession number CNA0002352 and CNA0002353).

Field-specific reporting

Please select the one below that is the best fit for your research. If you are not sure, read the appropriate sections before making your selection.

☒ Life sciences ☐ Behavioural & social sciences ☐ Ecological, evolutionary & environmental sciences

For a reference copy of the document with all sections, see nature.com/documents/nr-reporting-summary-flat.pdf

Life sciences study design

All studies must disclose on these points even when the disclosure is negative.

Sample size	Axenic cultures of Mesostigma viride (CCAC 1140) and Chlorokybus atmophyticus (CCAC 0220) were obtained from the Central Collection of Algal Cultures (CCAC; http://www.ccac.uni-koeln.de/) and grown in Waris-H culture medium (McFadden and Melkonian 1986). No statistical methods were used to predetermine sample sizes.
Data exclusions	The reads with low quality are more likely to contain errors, which might complicate the assembly process, and were thus excluded. To reduce the effect of sequencing error on assembly, we performed the quality control of raw data using the default parameters. Briefly, We used CLC Assembly Cell (version 5.0.1) to trim the adapters, remove duplicates and trim low quality bases. Then, we used Pairfq (version 0.16.0) (https://github.com/sestaton/Pairfq) to pair the reads. Finally, SOAPfilter (version 2.2) was used to filter the reads again. After filtering off duplicated reads, low quality reads and reads with adaptor sequences, 74.63 Gb and 14.51Gb high-quality clean reads remained for M. viride (CCAC1140) and C. atmophyticus (CCAC 0220), respectively, which were then subjected to a pipeline for genome assembly.
Replication	Since it was a Genome sequencing project, without any experiments. All the data were generated from a single axenic culture. Therefore, no replications were required.
Randomization	Since it was a Genome sequencing project, without any experiments. All the data were generated from a single axenic culture. Therefore, no randomizations were required.
Blinding	Since it was a Genome sequencing project, without any experiments. All the data were generated from a single axenic culture. Therefore, no blinding experiments were required.

October 2018

Reporting for specific materials, systems and methods

We require information from authors about some types of materials, experimental systems and methods used in many studies. Here, indicate whether each material, system or method listed is relevant to your study. If you are not sure if a list item applies to your research, read the appropriate section before selecting a response.

Materials & experimental systems

n/a	Involved in the study
☒	☐ Antibodies
☒	☐ Eukaryotic cell lines
☒	☐ Palaeontology
☒	☐ Animals and other organisms
☒	☐ Human research participants
☒	☐ Clinical data

Methods

n/a	Involved in the study
☒	☐ ChIP-seq
☒	☐ Flow cytometry
☒	☐ MRI-based neuroimaging
